# An Electrochemical Perspective of Aqueous Zinc Metal Anode

**DOI:** 10.1007/s40820-023-01227-x

**Published:** 2023-11-17

**Authors:** Huibo Yan, Songmei Li, Jinyan Zhong, Bin Li

**Affiliations:** School of Materials Science and Engineering, Beihang University, Beijing, 100191 People’s Republic of China

**Keywords:** Aqueous zinc ions batteries, Parasitic reactions, Aqueous electrolyte, Zinc anode

## Abstract

Based on the attributes of nonflammability, environmental benignity, and cost-effectiveness of aqueous electrolytes, as well as the favorable compatibility of zinc metal with them, aqueous zinc ions batteries (AZIBs) become the leading energy storage candidate to meet the requirements of safety and low cost. Yet, aqueous electrolytes, acting as a double-edged sword, also play a negative role by directly or indirectly causing various parasitic reactions at the zinc anode side. These reactions include hydrogen evolution reaction, passivation, and dendrites, resulting in poor Coulombic efficiency and short lifespan of AZIBs. A comprehensive review of aqueous electrolytes chemistry, zinc chemistry, mechanism and chemistry of parasitic reactions, and their relationship is lacking. Moreover, the understanding of strategies for suppressing parasitic reactions from an electrochemical perspective is not profound enough. In this review, firstly, the chemistry of electrolytes, zinc anodes, and parasitic reactions and their relationship in AZIBs are deeply disclosed. Subsequently, the strategies for suppressing parasitic reactions from the perspective of enhancing the inherent thermodynamic stability of electrolytes and anodes, and lowering the dynamics of parasitic reactions at Zn/electrolyte interfaces are reviewed. Lastly, the perspectives on the future development direction of aqueous electrolytes, zinc anodes, and Zn/electrolyte interfaces are presented.

## Highlights

- Detailed discussion and summary of aqueous electrolyte chemistry, parasitic reactions chemistry, and storage energy chemistry and their relationship in aqueous zinc ions batteries are conducted.
- The recent development of strategies for enhancing the inherent stability of electrolyte and zinc anode to restrain parasitic reactions is reviewed from a thermodynamic perspective.
- The regulation strategies of electrolyte/electrode interfaces to block parasitic reactions by adsorbents and solid electrolyte interphase are reviewed from a kinetic perspective.

## Graphical abstract

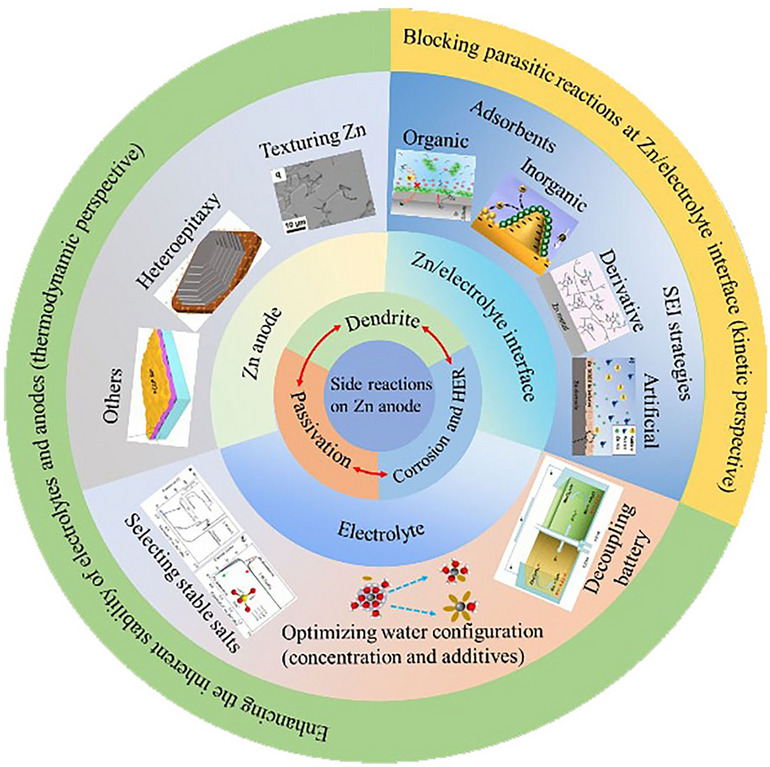

## Introduction

**Fig. 1:**
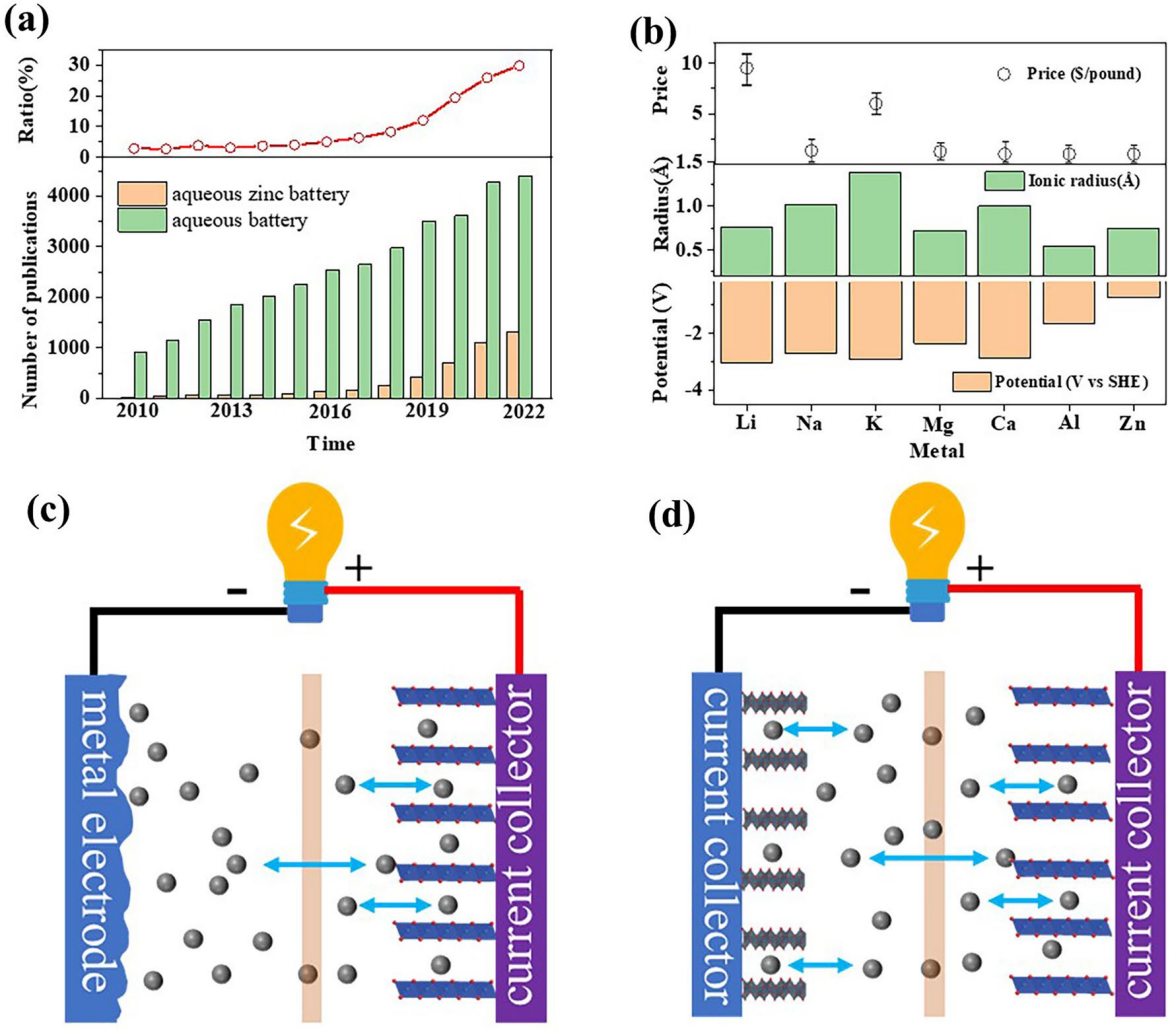
**a** Number of articles on AZIBs and aqueous batteries investigated from 2010 to November 2022 at Web of Science, and the proportion of published articles on AZIBs in aqueous batteries in each year. Search keywords including “aqueous battery” not “non-aqueous” for aqueous batteries search results. Search keywords including “aqueous zinc battery” or “aqueous zinc ion battery” or “aqueous zinc metal battery” not “non-aqueous” for AZIBs search result. **b** Comparison of the metal electrode potential, metal ion radius, and market price of the widely researched metal anodes. **c** and **d** Types of AMIBs based on anode classification. **c** Schematic illustration of the AMIBs with striping/plating anodes. Charge carriers (metal ions) are stripping from anode while intercalating into cathode or plating on anode, while de-intercalating from the cathode, represented by aqueous Mg, Ca, and Zn-ion batteries. **d** Schematic illustration of the AMIBs with intercalation/de-intercalation anodes. They are similar to the traditional rocking chair LIBs, represented by aqueous Li, Na, and K-ion batteries

In recent years, a series of electrochemical energy technologies, rechargeable batteries for storing electrical energy, have been extensively investigated and some of them have achieved great success in many aspects of modern life benefiting from the stable output and long cycle life, such as lead-acid and lithium-ion batteries (LIBs), but they are still not able to large-scale application due to the toxicity, pollution or high price. The necessary characteristics of batteries suitable for large-scale applications are safety, non-toxicity, low-cost, long cycle life, and high energy and power density. Considering these requirements, aqueous metal ions batteries (AMIBs), including Na^+^, K^+^, Zn^2+^, Ca^2+^, Mg^2+^, and Al^3+^, are attracting numerous researchers’ interest owing to the following shining advantages (Fig. 1a). Firstly, water (H_2_O) and the high abundance charge carriers render a low cost for AMIBs. Secondly, the non-toxic and nonflammable natures of solvent are fundamental for the safety of AMIB. Last but not least, aqueous electrolytes with a high ionic conductivity (aqueous electrolytes: > 50 mS cm^−1^, organic electrolytes: < 10 mS cm^−1^) enable a fast charge/discharge property [1–3].

In AMIBs, the extensively studied aqueous sodium, potassium, aluminum, magnesium, and calcium ion batteries are restricted by their fatal drawbacks. In aqueous sodium and potassium ions batteries, the intercalation type anode materials led to limited output potential, less available cathode materials, and underutilized storage capacity (Fig. 1d) [4–6]. In aqueous aluminum ion batteries, the strong electrostatic interaction between the Al^3+^ and the host lattice of cathode materials trigger sluggish kinetics and underutilized storage capacity [7]. Generally, magnesium and calcium can be directly used in aqueous batteries. Yet, the highly reactive metal electrodes as anodes (− 2.36 V vs. SHE for Mg; − 2.86 V vs SHE for Ca) inevitably continuously consume electrolyte and active metal, resulting in spanking hydrogen evolution reaction (HER), decreased Coulombic efficiency (CE), and poor lifetime (Fig. 1b, c) [8]. Fortunately, zinc metal shows high stability and compatibility in aqueous electrolytes due to the large overpotential for hydrogen evolution (despite its low redox potential − 0.76 V vs. standard hydrogen electrode (SHE)) (Fig. 1b). Additionally, the other advantages of Zn anode are particularly appealing: (i) two-electron redox property offers a high energy density (820 mAh g^−1^ and 5851 mAh cm^−3^); (ii) low price of Zn metal and its small ionic radius. Moreover, a wide range of cathode materials can be coupled with Zn metal, including Mn-based [9, 10], V-based [11, 12], Prussian blue analogs (PBAs) [13], Mo-based [14], organics [15, 16], and other viable candidates (e.g., Co_3_O_4_ [17], Bi_2_Se_3_ [18], etc.). The inherent advantages of zinc metal anodes and aqueous electrolytes, as well as the wide range of alternative cathodes, have sparked extensive research on AZIBs (Fig. 1a).

Even though AZIBs have a promising future, they are still in the infancy due to the uncontrollable parasitic reactions, including zinc dendrites, passivation, hydrogen evolution, and corrosion at anode side. These reactions are directly or indirectly caused by water molecules and zinc anode. Unlike non-aqueous LIBs, the absence of a self-formed protective layer on electrodes cannot isolate the solvent (H_2_O) from the electrodes in AZIB, leading to serious contact and collision between electrolytes and electrodes. Especially, during battery operation, hydrating zinc ions (Zn(H_2_O)_6_^2+^) de-solvate to gather water molecules at electrodes interfaces and bring about the occurrence of parasitic reactions. Therefore, aqueous electrolyte is a double-edged sword for AZIBs.

Recently, to inhibit or mitigate these parasitic reactions in AZIBs, many studies have been carried out to balance the advantages and disadvantages of aqueous electrolytes and some progress has been made. One approach involves enhancing the inherent thermodynamic stability of AZIBs, including both electrolytes and zinc anodes. For instance, to enhance the thermodynamic stability of electrolyte, the O−H bond in the water molecule is enhanced via adjusting coordination environments of water molecules. A specific example is the conversion of water clusters (network water and water molecules linked by hydrogen bonds) into solvent sheath of cations (electrostatic force between water molecules and ions) in water-in-salt electrolytes (WiSE). The other approach is to reduce the dynamic process of parasitic reactions by lowering the contact (or collision) frequency between precursors (such as H_2_O and anion) and electrodes. It is an undisputed fact that both parasitic reactions and battery reactions occur at the electrolyte/electrode interfaces. Therefore, from the perspective of dynamics, regulating electrolyte/electrode interfaces to block the active sites by adsorbents and artificial (or deriving) solid electrolyte interphase (SEI) can efficaciously minimize parasitic reactions.

Some excellent review papers have been published, focusing on summarizing the recent progress made in avoiding parasitic reactions [19–28]. Specifically, the strategies for inhibiting parasitic reactions from the electrolyte/electrode interfaces [27, 29, 30], additives [31], electrolytes [32, 33], anodes [34], and water [19, 20, 35] are reviewed. All of them mainly summarize the recent progress on electrodes or a certain component of the electrolytes in mitigating parasitic reactions. A *comprehensive* review of both the fundamental science underlying parasitic reactions and their inhibition strategies from thermodynamic (including electrolytes and anodes) and dynamic (electrolyte/electrode interfaces) perspectives is still desirable. This review aims to provide an understanding of the parasitic reaction chemistry, while summarizing the reported strategies to suppress these side reactions from thermodynamic and kinetic perspectives. We start with introducing the water and ions configurations in electrolyte and the thermodynamic stability of these configurations, and then, we introduce parasitic reactions at the anode side, and the relationships of parasitic reactions and composition configurations in electrolyte. In the following section, we will summarize the strategies to restrain parasitic reactions from the perspective of thermodynamics to enhance the inherent stability of electrolytes and anodes, and from the perspective of dynamics to lower the contact (or collision) frequency between precursors and electrodes at electrolyte/electrode interfaces. Lastly, we put forward our perspectives on overcoming the bottlenecks of zinc anode and promising direction for further development of high-performance AZIBs. We hope to provide a deep understanding on parasitic reactions chemistry in AZIBs and effective inspirations for mitigating parasitic reactions occurring in AZIBs.

## Aqueous Electrolyte Chemistry and Zinc Parasitic Reaction Chemistry in AZIBs

### Aqueous Electrolyte Chemistry

In this section, water solvent chemistry and solvation ion chemistry in aqueous electrolytes will be introduced.

#### Water Solvent Chemistry in Aqueous Electrolyte

**Fig. 2:**
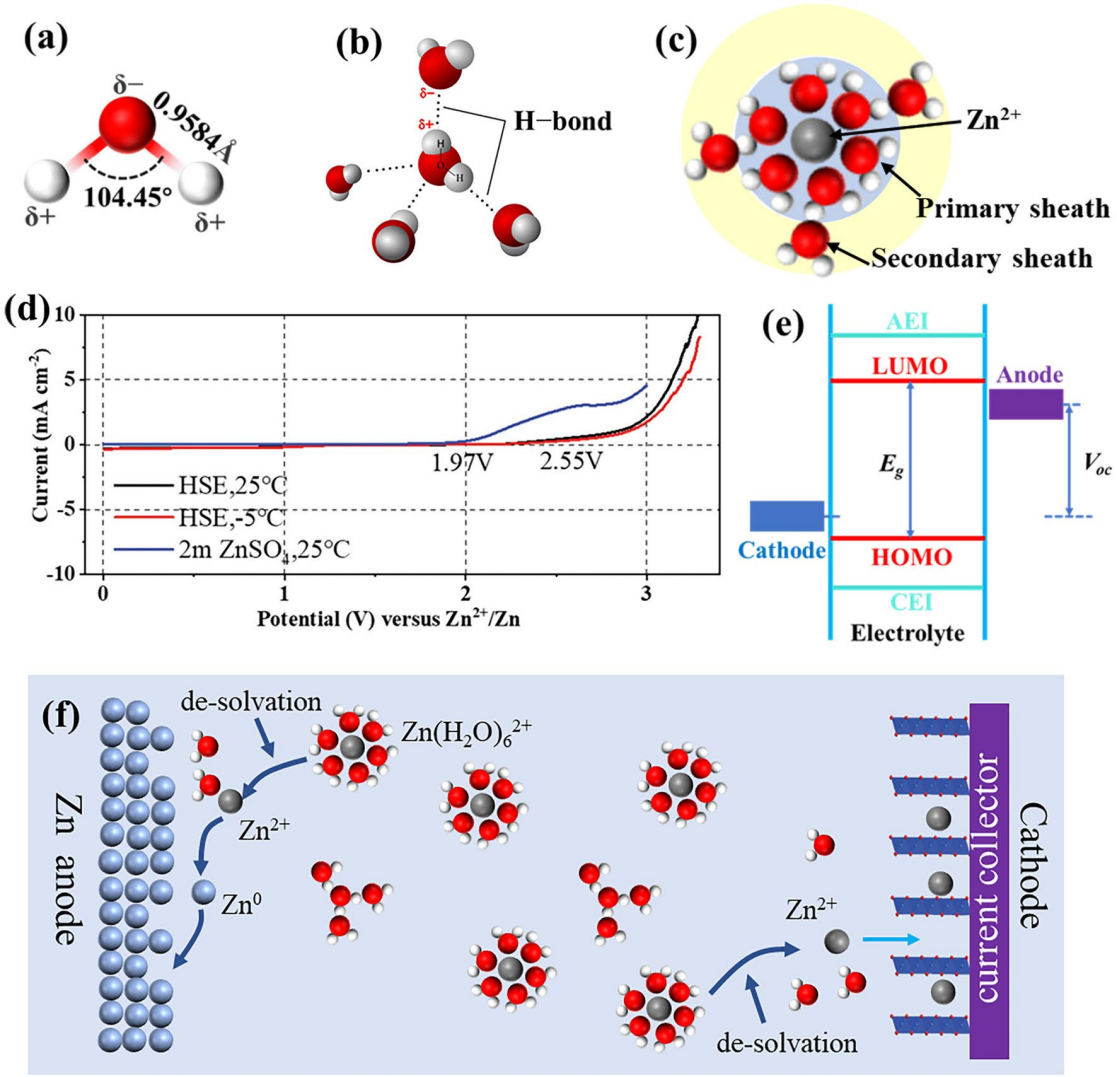
**a** Schematic illustration of water molecular structure. **b** Schematic illustration of water clusters formed through hydrogen bonds. **c** Schematic illustration of hydrated structure of zinc ions in conventional dilute aqueous electrolytes. **d** Oxygen onset potential in hydrated salt electrolyte (HSE) with different states and in a common dilute electrolyte (2 M ZnSO_4_). The HSE has different water configurations at 25 ℃ and -5 ℃. Reprinted with permission from Ref. [42]. Copyright 2022 John Wiley and Sons. **e** Band diagrams of the HOMO and LUMO of batteries with non-aqueous electrolytes. Cathode potential (*μ*_C_) should be higher than the HOMO of the electrolyte and anode potential (*μ*_A)_ could be lower than the LUMO. Otherwise, OER and HER will occur. The chemical potential difference between both electrodes is the open circuit voltage of the battery. **f** Schematic illustration of configuration structure of water molecules and zinc ions in electrolyte, the process of zinc ions incorporated into zinc lattice, and insertion of zinc ion into layered cathode

H_2_O, a polar molecule, contains a pair of no-collinear polar covalent bonds (O−H bonds), resulting in the oxygen atom end with negative charge and the hydrogen atom ends with positive charge (Fig. 2a) [36]. Hydrogen bond is formed by electrostatic interaction between the O atom of one H_2_O molecule and the H atom of its adjacent water molecules (Fig. 2b). In liquid water, multiple water molecules form a water cluster ((H_2_O)_n_) through hydrogen bonding [37]. Once soluble salts (such as ZnSO_4_) are added, the original hydrogen bond network is destroyed. Instead, water molecules are coordinated with ions through electrostatic force to reconstruct a thermodynamic stable state. Thus, individual Zn^2+^ ions do not exist but form hydrated Zn^2+^ (Zn(H_2_O)_n_^2+^) due to the polar nature of water [38]. As shown in Fig. 2c**,** in conventional dilute electrolytes, water molecules closely coordinate with Zn^2+^ through electrostatic force to form a primary solvation sheath (Zn(H_2_O)_6_^2+^) [39]. Then, the secondary solvation sheath is formed by hydrogen bonding between water molecules and the primary sheath [40]. The water cluster and solvated sheath structure in aqueous electrolytes have been widely confirmed by electrochemical experiments, Fourier infrared spectroscopy (FT-IR), Raman spectroscopy, nuclear magnetic resonance, and molecular dynamics simulation [41–48]. In aqueous electrolytes, various water configuration is in dynamic equilibrium, such as the dynamic equilibrium transformation of water clusters and single water molecules (Eq. 1), and ionization equilibrium of free water (Eq. 2). The single free water molecule tends to polymerize into water clusters or solvate cations. Therefore, the concentration of single free water molecule is extremely low because of the unstable state in thermodynamics [49]. In short, the water molecular configurations in electrolytes include water clusters (hydrogen bonds between water molecules), solvent sheath water molecules (electrostatic force between water molecules and ions), and a trace of independent water molecules.

1 $$\left( {{\text{H}}_{{2}} {\text{O}}} \right)_{{\text{n}}} { \leftrightharpoons }\left( {{\text{H}}_{{2}} {\text{O}}} \right)_{{{\text{n}} - {1}}} + {\text{H}}_{{2}} {\text{O}}$$

2 $${\text{H}}_{{2}} {\text{O}} \rightleftharpoons {\text{H}}^{ + } + {\text{OH}}^{ - } \left( {{\text{Kw}} = {\text{c}}\left( {{\text{H}}^{ + } } \right) \times {\text{c}}\left( {{\text{OH}}^{ - } } \right) = {1} \times {1}0^{{ - {14}}}\, {\text{at 25 }}{^\circ } {\text{C}}} \right)$$

The thermodynamic stability of water, an inherent property, is directly influenced by the strength of the O–H bond. Importantly, different configurations of water exhibit varying strengths of the O–H bond. Therefore, water clusters, solvent sheath water molecules, and individual water molecules exhibit differential thermodynamic stability properties. For example, a hydrated salt electrolyte suppresses the presence of water cluster and elevates the decomposition voltage to 2.55 V versus Zn^2+^/Zn. However, the 2 M ZnSO_4_ shows a low decomposition voltage of 1.98 V versus Zn^2+^/Zn, indicating the formation of hydrogen bond reduces the thermodynamic stability of water molecules (Fig. 2d) [42]. Thus, many works have been done to enhance the thermodynamic stability of electrolytes by reducing the hydrogen bond density of water molecules, such as water-in-salt electrolytes (WiSE), hydrated salt electrolytes, and deep eutectic electrolytes.

#### Solvation Zinc Ion Chemistry in Aqueous Electrolyte

Zn(H_2_O)_6_^2+^, as current conductor in the electrolyte, migrates between the cathode and anode to form a circuit under the presence of electric field to realize the storage and release of energy. Specifically, the conversion of Zn^2+^ ↔ Zn^0^ at Zn anode (Eq. 3) and the insertion/extraction of Zn^2+^ at cathode are carried out. However, Zn^2+^ exists in the form of solvated Zn^2+^ (Zn(H_2_O)_6_^2+^) rather than as individual ions in the electrolyte. As a result, the solvated zinc ions (Zn(H_2_O)_6_^2+^) locating at electrodes interfaces need to de-solvate and release water molecules (Eq. 4). In detail, during the charging process, Zn(H_2_O)_6_^2+^ de-solvates into Zn^2+^ and is reduced and incorporated into the zinc lattice at the anode, while Zn^2+^ is extracted from the cathode to assemble into Zn(H_2_O)_6_^2+^. Conversely, during the discharge process, zinc atoms (Zn^0^) are oxidized to zinc ions (Zn^2+^) to assemble into Zn(H_2_O)_6_^2+^ at anode interface, while Zn(H_2_O)_6_^2+^ de-solvates to Zn^2+^ and inserts into cathode (Fig. 2f). Thus, a variable environment is experienced at electrode surface during charging/discharging process.

3 $${\text{Zinc anode: Zn}}^{{2 + }} + 2{\text{e}}^{ - } \leftrightarrow {\text{Zn}}^{0}$$

4 $${\text{De-solvation}}\,{\text{process: Zn}}\left( {{\text{H}}_{2} {\text{O}}} \right)_{6}^{{2 + }} \to {\text{Zn}}^{{2 + }} + 6{\text{H}}_{2} {\text{O}}$$

The independent water molecules from the de-solvation of Zn(H_2_O)_6_^2+^ are close to electrode surfaces. Compared with the water clusters and solvent sheath water molecules, the independent free water molecules show poor stability in thermodynamics, thus leading to serious parasitic reactions on electrodes interface. The stability of electrode interfaces is highly related to the electrode/electrolyte interface, composing of cathode-electrolyte interface (CEI) and anode electrolyte interface (AEI) [50]. For organic LIB (Fig. 2e), the maximum operating voltage range of a full cell is the difference between the energy of the lowest unoccupied molecular orbital (LUMO) and the highest occupied molecular orbital (HOMO); *μ*_A_ and *μ*_C_ are electrochemical potentials of the anode and cathode, respectively. When *μ*_A_ is higher than the LUMO energy, electrolyte solvent is reduced to form AEI; similarly, if the *μ*_C_ is lower than the HOMO energy, electrolyte solvent is oxidized to form CEI, respectively. The AEI and CEI prevent the further occurrence of parasitic reactions. Nevertheless, the absence of AEI and CEI films in AZIBs; instead, parasitic reactions involving electrolytes occur at anode and cathode.

In short, in the operation of AZIBs, a variable solvent environment, the poor stability of electrolytes, and the absence of AEI and CEI films promote serious parasitic reactions at electrodes interface. The activity order of water configuration in electrolytes is independent water > water cluster > solvated water. Understanding and adjusting the water molecular chemistry in AZIBs is an effective breakthrough for building stable AZIBs. In the next section, we will introduce the mechanism of parasitic reactions and their relationship.

### Zinc Parasitic Reaction Chemistry in AZIBs

During the operation of AZIBs, the de-solvation of hydrated zinc ions occurs at the electrode interface, which changes the local environment and leads to various side reactions. It is necessary to gain a deeper understanding of the source of parasitic reactions and find effective measures to mitigate them. The parasitic reactions in AZIBs include corrosion, passivation, and dendrites on zinc anode. In this section, we will delve into the mechanism of these parasitic reactions.

#### Zinc Corrosion

**Fig. 3:**
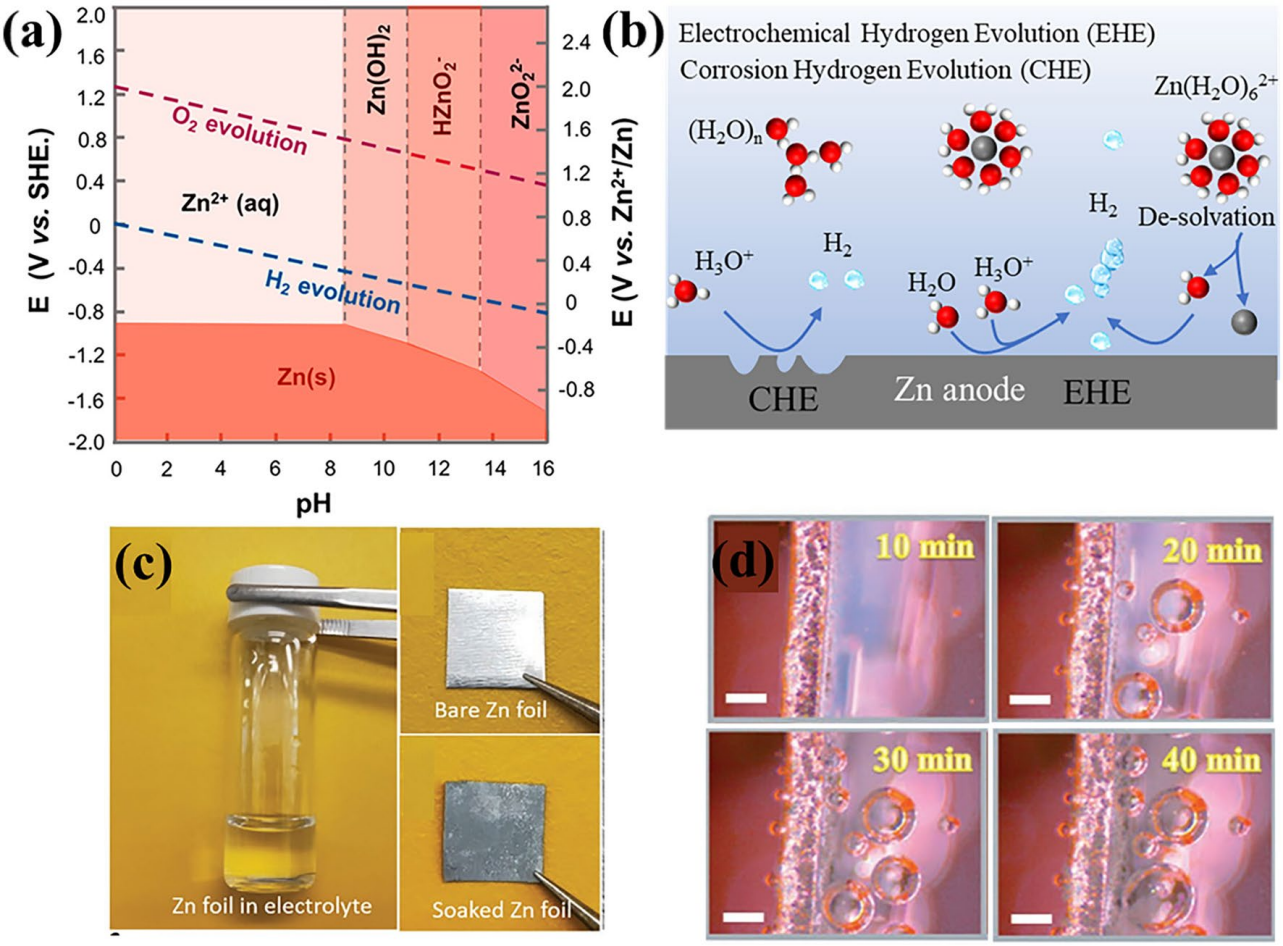
**a** Pourbaix diagram of water and zinc with the y-axis of potentials (V vs. SHE) and the x-axis of pH values. The area between the red and blue lines represents the upper and lower thermodynamically stable potentials as a function of pH values. The thermodynamically stable line of zinc (red line) is lower than the water reduction (HER) line, indicating the thermodynamic instability characteristics of zinc metal in aqueous electrolyte. Reprinted with permission from Ref. [59]. Copyright 2020 American Chemical Society. **b** Schematic illustration of corrosion hydrogen evolution and electrochemical hydrogen evolution. **c** Photographs of a zinc foil before and after soaking in 1 M ZnSO_4_ electrolyte. Proof of self-corrosion of zinc in electrolyte. Reprinted with permission from Ref. [57]. Copyright 2020 John Wiley and Sons. **d** In situ optical visualization observations of Zn plating at 10 mA cm^–2^. Proof of the existence of electrochemical hydrogen evolution and dendrites. Reprinted with permission from Ref. [58]. Copyright 2021 John Wiley and Sons

Zinc is prone to corrosion in acidic and alkaline environments and undergoes hydrogen evolution reactions. The pH values of commonly used mildly acidic electrolytes are 3∼7 [51]. The acidic nature of the electrolytes can be attributed to the following two facts. Firstly, the OH^─^, which is generated by the ionization of water, complexes with Zn^2+^ (Eq. 6), leading to an increase in the concentration of H^+^. As we all know, the water in the electrolyte is ionized with a constant concentration product (*K*_w_) at constant temperature (Eq. 5). Once the concentration of OH^─^ (c(OH^─^)) decreases, the concentration of H^+^ becomes dominant. Secondly, due to the polar nature of water, the coordinated water molecules in the Zn(H_2_O)_6_^2+^ species were hydrolyzed to produce H^+^ [52, 53]. Thus, the pH of the electrolyte shows acidity (pH < 7). From the Pourbaix diagram of zinc **(**Fig. 3a), metal zinc shows thermodynamic instability in acid electrolytes [54]. Due to the absence of AEI [55], the displacement reaction will occur on zinc anode (Eq. 7) that H^+^ will be reduced to H_2_ and Zn^0^ will be oxidized to Zn^2+^ [56]. This process is called corrosion hydrogen evolution (CHE), which can take place during the charging, discharging, and idling processes of AZIBs (Fig. 3b). As shown in Fig. 3c**,** the zinc foil soaked in 1 M ZnSO_4_ electrolyte for a week is corroded and tarnished [57]. This displacement should trigger invalid consumption of zinc and battery expansion. It should be noted that the CHE rate is very slow and will last for a long time [24].

5 $${\text{H}}_{{2}} {\text{O}} \rightleftharpoons {\text{H}}^{ + } + {\text{OH}}^{ - } \left( {{\text{Kw}} = {\text{c}}\left( {{\text{H}}^{ + } } \right) \times {\text{c}}\left( {{\text{OH}}^{ - } } \right) = {1} \times {1}0^{{{-14}}} {\text{at 25}}{^\circ } } {\text {C}}\right)$$

6 $${\text{Zn}}^{{{2} + }} + {\text{OH}}^{ - } \rightleftharpoons {\text{Zn}}\left( {{\text{OH}}} \right)^{ + }$$

7 $${\text{Zn}}^{0} + {\text{2H}}^{ + } \to {\text{Zn}}^{{{2} + }} + {\text{H}}_{{2}}$$

During Zn deposition process, zinc electrode has a more negative potential to overcome the de-solvation energy barrier. The removed water molecules from the solvent sheath accumulate on zinc anode surface (Fig. 3b), which are more likely to obtain electrons on the zinc metal surface to generate H_2_ and OH^─^ (Eqs. 8 and 9). Therefore, electrochemical hydrogen evolution (EHE) is further triggered. The severe EHE process resulted in extremely alkaline microzones, which in turn led to severe zinc corrosion (Eq. 10). As shown in Fig. 3d**,** an in situ optical visualization observation is built to monitor electrochemical hydrogen evolution and zinc dendrites [58]. Abundant gas bubbles are observed within 20 min, and the bubbles volume gradually increases with time.

8 $${\text{2H}}^{ + } + {\text{2e}}^{ - } \to {\text{H}}_{{2}}$$

9 $${\text{2H}}_{{2}} {\text{O}} + {\text{2e}}^{ - } \to {\text{H}}_{{2}} + {\text{2OH}}^{ - }$$

10 $${\text{Zn}} + {\text{2OH}}^{ - } + {\text{2H}}_{2} {\text{O}} \to {\text{Zn}}\left( {{\text{OH}}} \right)_{4}^{2-}\, + {\text{H}}_{{2}}$$

Hazards of zinc corrosion mainly include: (1) the electrolyte solvent is exhausted; (2) the battery volume expands to lead to cell rupture; (3) a reduced Coulombic efficiency is obtained; (4) metal zinc is consumed (and corroded); (5) the formation of passivation products and dendrites are promoted. The suppression of HER is a difficult problem that must be overcome to realize the wide application of zinc ion batteries.

#### Zinc Passivation

The HER process in microregion consumes H^+^ and generates OH^─^, resulting in the accumulation of a high concentration of OH^─^ [60]. This alkaline microregion, encountered by zinc ions, anions, and free water molecules, leads to the formation of passivation products (Eq. 10). In fact, when the zinc foil is immersed in electrolytes for a long time, the corresponding passivation products (Zn_4_SO_4_(OH)_6_ xH_2_O, Zn_4_ClO_4_(OH)_7_) are also observed on zinc surface [56, 57], indicating that CHE can also contribute to the formation of passivation products. These passivation products are loosely stacked and cannot prevent water from attacking zinc anode. Moreover, these passivation films cannot facilitate the conduction of zinc ions.

At present, various alkaline zinc salt products are found in the corresponding electrolyte salt [56, 61–63]. The accumulation of passivation products on zinc anode causes several issues. Firstly, it increases the overpotential of zinc ion deposition, thereby accelerating the HER process; Secondly, it promotes uneven zinc ion deposition, which accelerates dendrite growth; Thirdly, it results in the uncontrolled consumption of electrolyte components, including salt and water, consequently reducing the ionic conductivity.

Alkaline salt precipitation (taking ZnSO_4_ electrolyte as an example):

11 $${\text{4Zn}}^{{{2} + }} + {\text{6OH}}^{ - } + {\text{SO}}_{{4}}^{{{2} - }} + {\text{xH}}_{{2}} {\text{O}} = {\text{ZnSO}}_{{4}} \left[ {{\text{Zn}}\left( {{\text{OH}}} \right)_{{2}} } \right]_{{3}} \cdot{\text{xH}}_{{2}} {\text{O}}$$

#### Zinc Dendrites

**Fig. 4:**
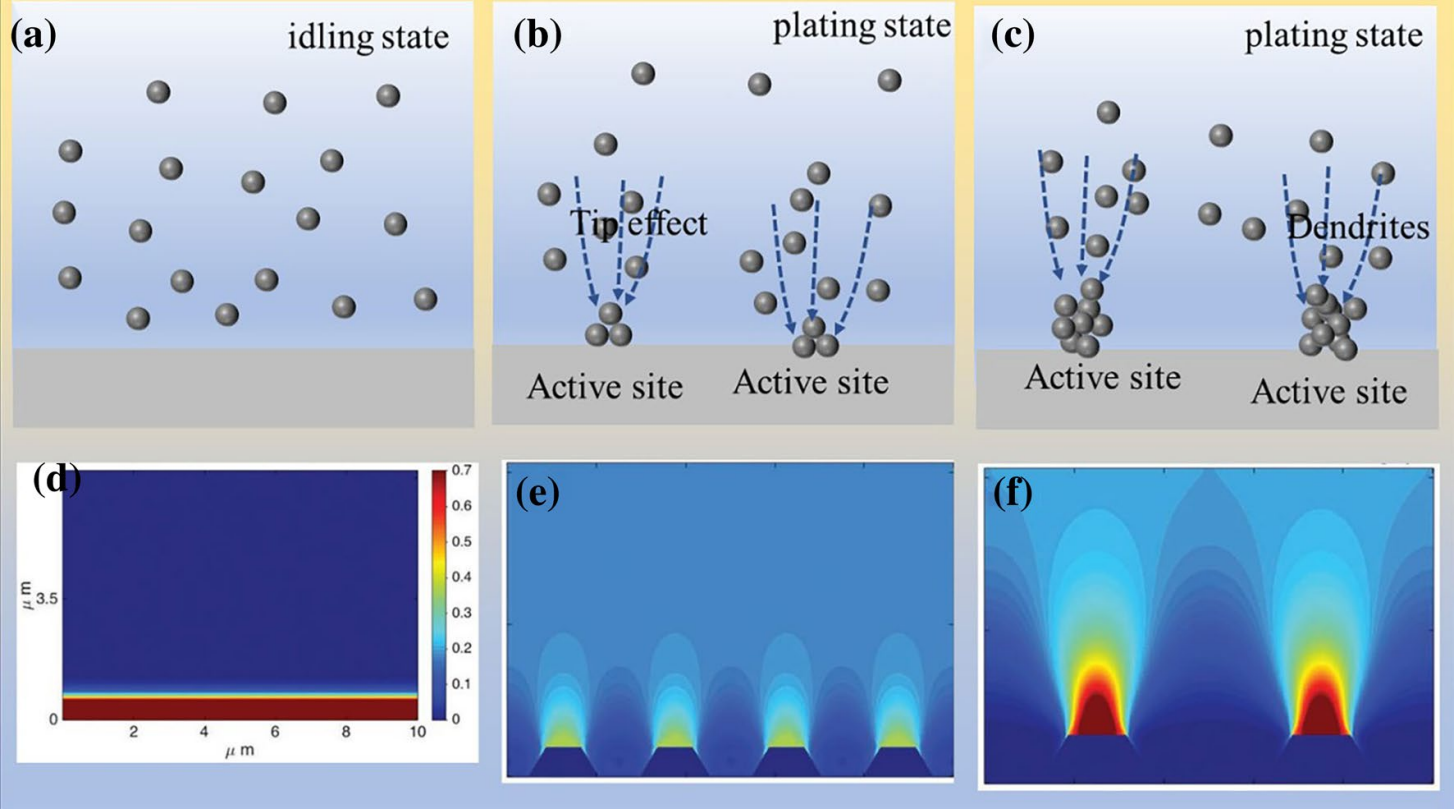
Schematic diagram of dendrite formation. **a** Uniform distribution of zinc ions under idling state. **b** Evolution and growth process of deposition site (active site). **c** Dendrites are initiated by uneven deposition. Simulate the zinc ions distribution along different dendritic sizes: **d** flat surface, **e** small dendritic seeds, **f** large dendritic seeds. Reprinted with permission from Ref. [58]. Copyright 2019 John Wiley and Sons

Similar to other metal batteries, the zinc metal anode operates based on an ion deposition/dissolution mechanism. The deposition/dissolution rates of zinc anode are primarily controlled by the mass transfer of zinc ions in the liquid phase, and the influence factors have been widely studied, such as zinc surface texture, electric field distribution, and salt concentration. The formation principle of zinc dendrites is shown in Fig. 4a–c. In the idle state of the battery system, the distribution of zinc ions in the electrolyte is relatively uniform (Fig. 4a). Once zinc ion moves to the zinc electrode surface under an external electric field, they preferentially deposit at energetically active sites (such as grain boundary and defects), forming small protuberances [65]. Subsequently, based on the tip effect, a strong electric field distribution is generated at protuberances, which induces more zinc ions to gather at the active site and more uneven distribution of zinc ions in the electrolyte (Fig. 4b). It should be noted that this may lead to further concentration polarization, exacerbating the non-uniform mass transfer of zinc ions in the liquid phase. As deposition continues, the tip effect amplifies at the initial deposition sites, leading to greater unevenness in ion concentration, electric field distribution, and deposition rate, ultimately resulting in the formation of dendrites (Fig. 4c). Due to the hexagonal close-packed crystal structure, the zinc dendrites present 2D hexagonal plates. In practice, the morphology depends on the cyclic parameters [66]. The zinc dendrites progressively grow and enlarge, posing a risk of puncturing the porous separator and causing short circuits. What's more, zinc dendrites can detach from the root and free into the electrolyte, becoming "dead zinc" and significantly reducing the utilization rate of zinc. Consequently, the uncontrollable growth of zinc dendrites has become a major obstacle in the development of AZIBs.

Computer simulations have provided evidence for the uneven deposition rate and distribution of zinc ions. As shown in Fig. 4d–f, the distribution of the zinc ion concentration field is analyzed on substrates with different dendrite sizes [64]. It is clearly observed that when large dendrites are present on the substrate, the distribution of zinc ion concentration becomes highly uneven. The concentration of zinc ions is higher at positions with high electric field strength, such as the dendrite tips, while it is lower at positions with lower electric field strength, such as flat areas. In contrast, when the substrate lacks protrusions, the distribution of ion concentration becomes more uniform. These simulation results align with the mechanism of zinc dendrite formation resulting from uneven deposition. Zinc dendrites tend to become active sites for HER and zinc passivation due to the high interface energy. Therefore, the occurrence of parasitic reactions and the generation of zinc dendrites mutually reinforce each other. Several insightful reviews have focused on the formation and inhibition of zinc dendrites [59, 67–69]. In this review, we only regard zinc dendrites as active sites for parasitic reactions.

### Relationship of Electrolyte Chemistry and Zinc Parasitic Reaction Chemistry

**Fig. 5:**
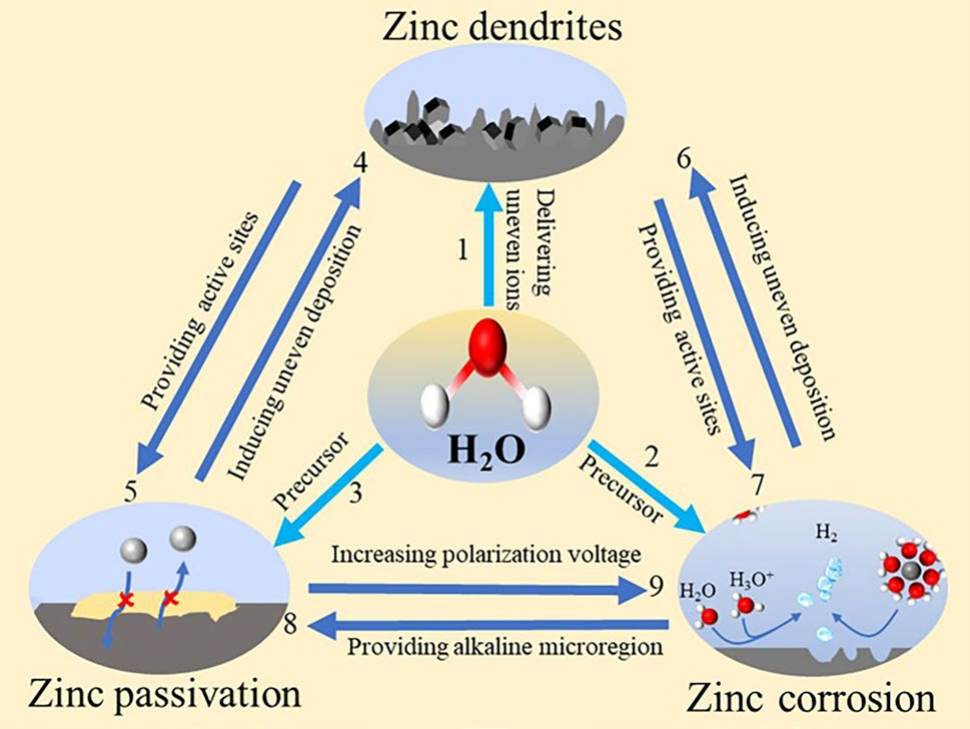
Schematic diagram of the relationship among zinc dendrite, passivation, and corrosion, and electrolyte

As described in the previous section, the uneven electric field distribution and morphological variations on zinc interface, and serious concentration polarization in electrolyte contribute to the non-uniformity transfer of zinc ions. During the deposition process, the electrolyte facilitates an uneven zinc ions flow, promoting the serious growth of dendrites (labeled as 1 in Fig. 5). As the precursors, electrolyte directly participate in HER and passivation reaction process (labeled as 2 and 3 in Fig. 5). For example, the components of alkaline salt precipitation (passivation by-products, ZnSO_4_[Zn(OH)_2_]_3_·xH2O) contain structural water molecules and OH^─^. Hence, the dendrite, passivation, and HER are closely intertwined with the electrolyte.

The disordered growth of dendrites increases the contact area between the zinc electrode and the electrolyte, providing more active sites for the occurrence of parasitic reactions, thereby accelerating the rate of parasitic reactions (labeled as 5 and 7 in Fig. 5). Conversely, the parasitic reactions promote zinc ions uneven deposition to accelerate dendritic growth. For example, passivation by-products, which do not conduct zinc ions, accumulate on the surface of the zinc electrode, causing uneven deposition and accelerating dendrite growth (labeled as 4 in Fig. 5). HER disrupts the smoothness of the zinc surface and accelerates dendrite growth (labeled as 6 in Fig. 5). Note that the growth of zinc dendrites is accompanied by the de-solvation of Zn(H_2_O)_6_^2+^ on zinc surface to generates active water molecules, which can attack the zinc electrode to cause parasitic reactions. HER provides an alkaline microregion for the zinc passivation, which promotes the formation of by-products (labeled as 8 in Fig. 5). The formation of passivation products consumes OH^─^ and further promotes HER. More seriously, the accumulation of by-products on zinc surface increases the electrochemical overpotential to facilitate HER (labeled as 9 in Fig. 5). In conclusion, the relationship among dendrites, passivation, and HER is relevant. Therefore, it can effectively inhibit dendrites by regulating the activity of water molecules and electrodes to avoid the occurrence of parasitic reactions.

The advanced and reasonable characterization techniques to understand the parasitic reactions for boosting the development of AZIBs toward practical applications are necessary. Thus, massive reviews have focused on the ex‐situ and in situ techniques to observe parasitic reactions [70–72]*.* Especially, some complex transient parasitic reactions and continuous information, for example, the zinc dendrites forming-growing kinetics, can observe and track electrode degradation in real‐time by in situ characterizations. Based on the recent review articles on characterization of parasitic reactions, this review will not report the ex situ and in situ techniques.

The parasitic reactions and batteries reactions in AZIBs are influenced by both of electrolytes and electrodes. One reasonable method to suppress parasitic reactions is to enhance the thermodynamic stability of battery components (zinc anodes and electrolytes), which is the inherent properties and independent of the battery environment and states. Another method is to avoid or reduce the contact between electrolytes and electrodes from a kinetic perspective, which adjusts the electrolyte/electrode interfaces to avoid parasitic reactions. In the following two sections, strategies for suppressing parasitic reactions in AZIBs will be reviewed from the perspectives of enhancing inherent stability of electrolytes and zinc anodes (Sect. 3), and restraining the kinetics of parasitic reactions (Sect. 4).

## Enhancing the Inherent Stability of Battery Modules

The stability of electrolytes primarily depends on the solvent molecules and the composition of salt. As previously mention (Sect. 2.1), the configuration structure of water molecules in traditional dilute electrolytes includes water clusters, solvent sheath water molecules, and a trace of independent water molecules. Of note, the configuration structures directly determine the strength of the O−H bond in H_2_O molecules, which is an indicator of the thermodynamic stability of water. The activity order of water configurations in electrolytes is as follows: independent water > water cluster > solvated water. Independent water molecules are mainly accumulated at electrode interfaces during the de-solvation process of hydrated zinc ions. However, the generation of the highly active independent water molecules is unavoidable during AZIBs operation, unless the water sheath of zinc ion is completely replaced by non-water molecules. Water cluster molecules, compared to solvated water molecules, exhibit lower thermodynamic stability due to the weakening of the O−H bond strength caused by hydrogen bonding. In addition to the water solvent, the type of salt also affects the thermodynamic stability of the electrolyte. Some anions of zinc salt promote the occurrence of parasitic reactions. Strategies for improving the inherent stability of electrolytes to inhibit parasitic reactions are described in detail in Sect. 3.1.

Based on the different atomic arrays on various crystal facets, different crystal facets endow distinct physical, chemical, and thermodynamic stability properties. For example, Zn(002) as the densest packing surface shows minimum surface free energy, which is conducive to resisting parasitic reactions on zinc anode. Strategies for improving the inherent stability of zinc electrodes will be introduced in Sect. 3.2.

### Strategies for Enhancing the Inherent Stability of the Electrolyte

The composition of the electrolytes, including additives and zinc salts, can have a profound impact on the thermodynamic stability of electrolytes. Hence, it is multimodal to regulate the stability of electrolytes and to avoid parasitic reactions. In this section, we will summarize and discuss relevant thermodynamic mechanisms in latest progress, including (1) selecting appropriate zinc salts, in which different anions exhibit different thermodynamic stability (Sect. 3.1.1); (2) regulating water configuration via concentrating electrolyte (Sect. 3.1.2) and additives engineering (Sect. 3.1.3), which can break the hydrogen bond network of water clusters, arrest water molecules in cation solvation sheath, and reinforce the strength of O−H bond in water molecules.

#### Selecting Stable Zinc Salts for Enhancing Electrolyte Stability

**Fig. 6:**
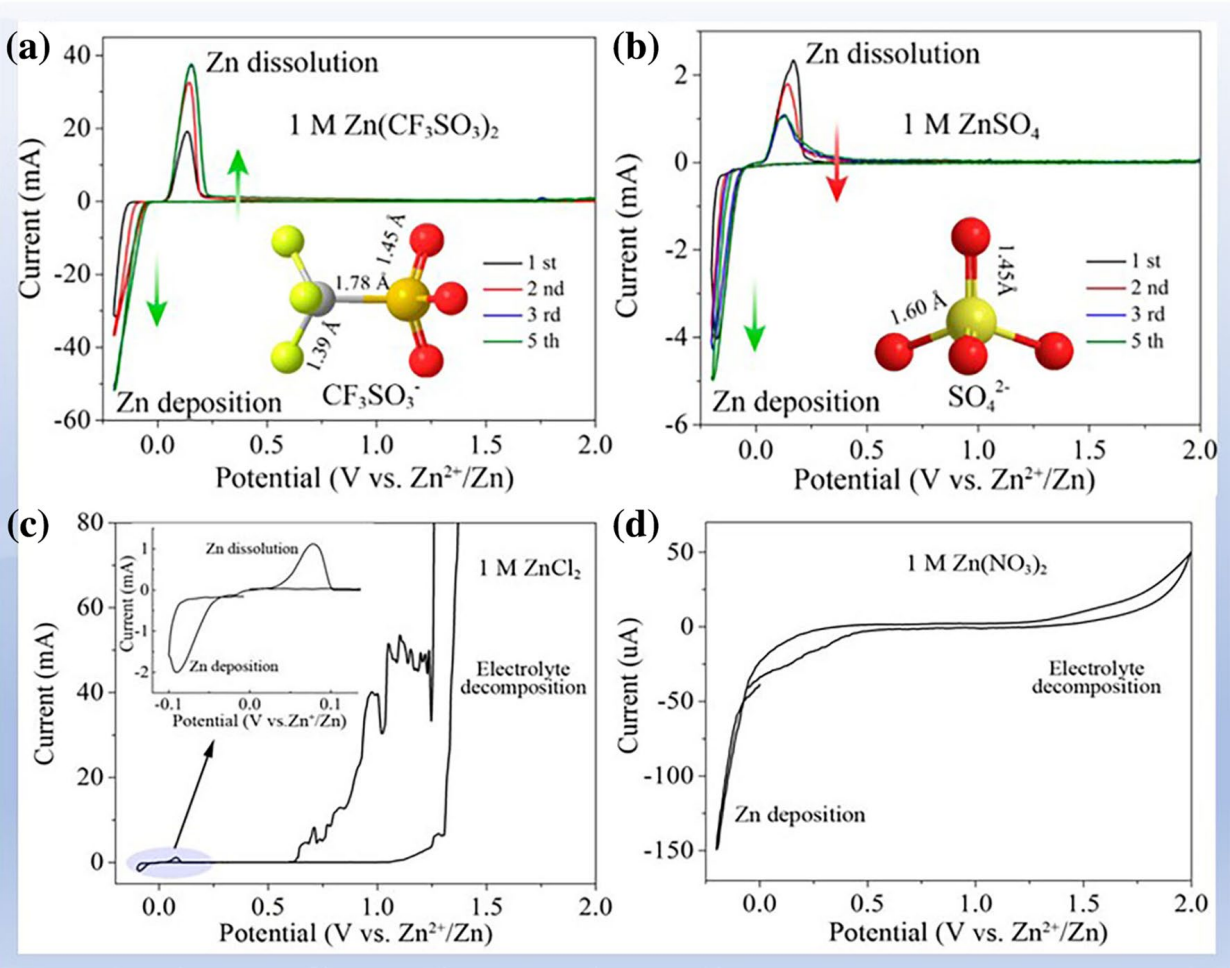
Zinc salt affecting electrolyte stability. **a** 1 M Zn(CF_3_SO_3_)_2_; **b** 1 M ZnSO_4_; **c** 1 M ZnCl_2_; and **d** 1 M Zn(NO_3_)_2_. Reprinted with permission from Ref. [62]. Copyright 2016 American Chemical Society

The types of salts are also critical to the thermodynamic stability of electrolytes. With the development of aqueous AZIBs, a series of zinc salts have been employed in electrolyte such as ZnSO_4_ [51], Zn(ClO_4_)_2_ [73, 74], ZnF_2_ [75], ZnCl_2_ [61, 76], Zn(CF_3_SO_3_)_2_ [51], Zn(CH_3_COO)_2_ [77], and Zn(TFSI)_2_ [78]. As shown in Fig. 6, the CVs of four zinc salts of Zn(CF_3_SO_3_)_2_, ZnSO_4_, ZnCl_2_, and Zn(NO_3_)_2_ electrolytes exhibit a distinguishable electrochemical behavior, indicating a differential thermodynamic stability. Specifically, the 1 M Zn(CF_3_SO_3_)_2_ and 1 M ZnSO_4_ show high voltage resistance potential to inhibit OER (2.4 and 2.3 V), while 1 M ZnCl_2_ and 1 M Zn(NO_3_)_2_, respectively, exhibit low OER onset potential (< 1.6 V) and serious HER, which are associated with the roles of anions. Due to the penetration function of Cl^−^ [79], a narrow anodic potential window and low CE are caused (Fig. 6c) [62]. In addition, NO_3_^−^ anions as strong oxidants can oxidize zinc foil and cause serious corrosion of the zinc anode (Fig. 6d) [75]. Therefore, dilute ZnCl_2_ and Zn(NO_3_)_2_ electrolytes are rarely used in AZIBs because of their poor thermodynamic stability [62, 76]. In contrast, 1 M ZnSO_4_ and 1 M Zn(CF_3_SO_3_)_2_ deliver a high CE and a wide electrochemical window (Fig. 6a, b) because of the stable structure of SO_4_^2−^ and the bulky volume of CF_3_SO_3_^−^ [62]. Moreover, compared with the ZnSO_4_ electrolyte, bulky CF_3_SO_3_^−^ can coordinate with zinc ion to reduce the number of water molecules in the solvent shell of Zn^2+^, alleviate parasitic reactions on the electrode surface, and enhance the stability of the electrolyte [39, 62]. In recent years, a similar coordination configuration of anion coordinated with Zn^2+^ is observed in Zn(CH_3_COO)_2_ and Zn(TFSI)_2_, based on the bulky volume of CH_3_COO^−^ and TFSI^−^. It should be emphasized that the high cost and complicated preparation process of Zn(CF_3_SO_3_)_2_ and Zn(TFSI)_2_ limited their wide application in AZIBs. A 1 m Zn(ClO_4_)_2_ electrolyte shows good anodic stability up to 2.4 V and a high CE of 99.0% based on the stable containing Cl^−^ protective layer on Zn anode, which originates from trace the reduction in ClO_4_^−^ during battery operation [73]. However, Zn^2+^ may coordinate with ClO_4_^−^ to produce ZnO passivation layer on zinc anode, which increases the diffusion barrier of Zn^2+^ and causes a slow reaction kinetics [75, 80]. Due to the low solubility of ZnF_2_ (1.6 g/100 mL water at 20 °C), the solvent exists in the form of water clusters, so the ZnF_2_ electrolyte shows poor thermodynamic stability and is rarely used in zinc ion batteries up to now.

In summary, the future market of electrolytes for AZIBs will still be dominated by zinc salts with excellent compatibility with various cathodes and zinc anodes, low price, and high thermodynamic stability, such as ZnSO_4_ and Zn(ClO_4_)_2_ [1].

#### Concentrating Electrolyte for Enhancing Electrolyte Stability

**Fig. 7:**
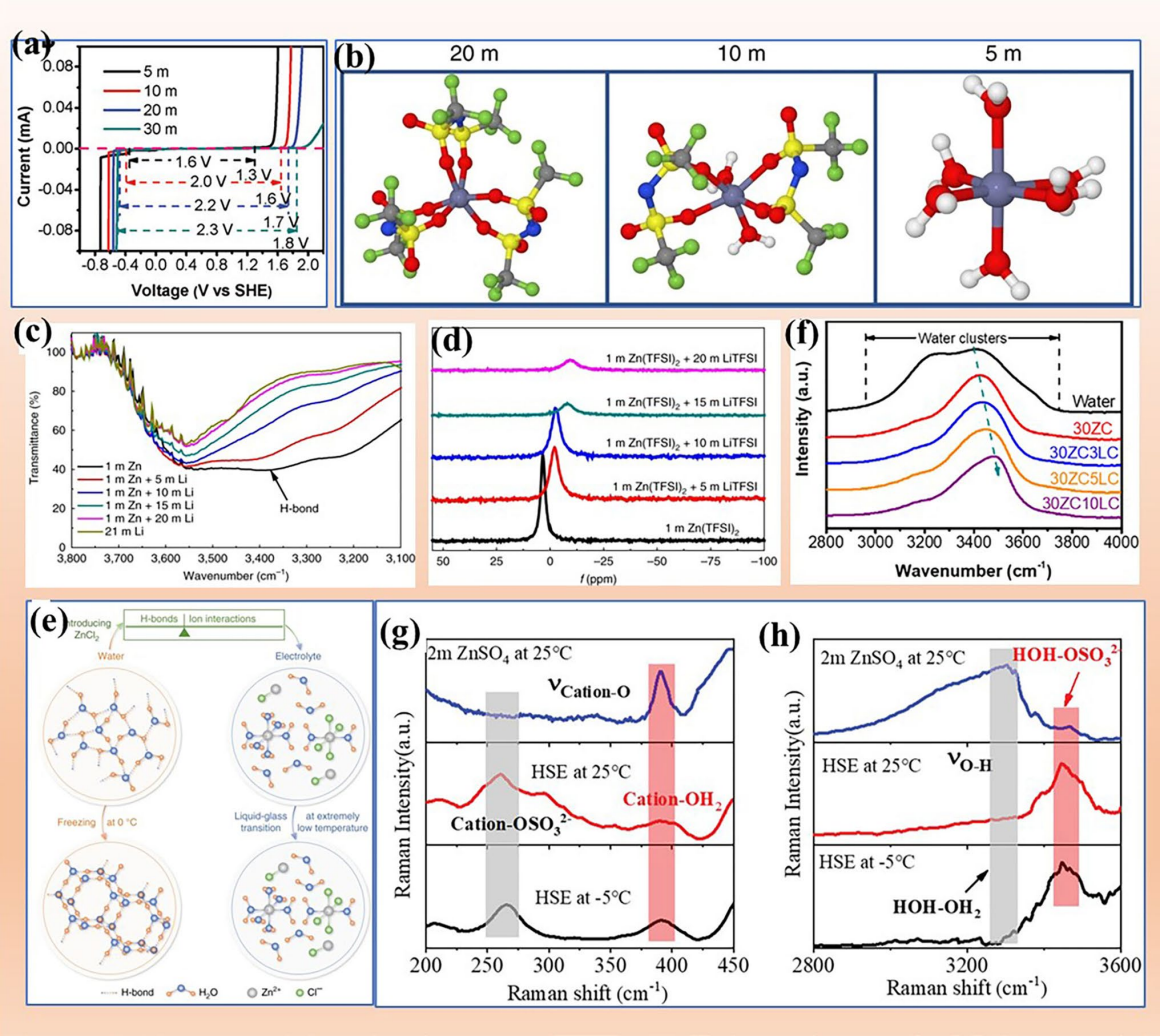
Concentrated salt improving electrolyte stability. **a** Electrochemical stability window of the ZnCl_2_ electrolyte with different concentrations. Reprinted with permission from Ref. [76]. Copyright 2018 The Royal Society of Chemistry. **b** Zn^2+^-solvation structure in 1 m Zn(TFSI)_2_ + Xm LiTFSI (X = 5, 10, and 20). **c** FTIR spectra and **d** ^17^O (water) NMR of the electrolyte with increasing salt concentration. Reprinted with permission from Ref. [39]. Copyright 2018 Springer Nature. **e** The structure evolution of water and electrolyte. At 0 °C, the original water network connected by hydrogen bond is easily transformed into ice network. The addition of ZnCl_2_ destroyed the hydrogen bond network and enhanced the ion interaction. Reprinted with permission from Ref. [93]. Copyright 2020 Springer Nature. **f** Femtosecond stimulated Raman spectroscopy (FSRS) of ZnCl_2_/LiCl mixture electrolytes. Reprinted with permission from Ref. [89]. Copyright 2020 John Wiley and Sons. **g** and **h** Raman spectroscopy of 2 M ZnSO_4_, and hydrated salt electrolyte. **g** Cation-O stretch, **h** O–H stretch vibration. Reprinted with permission from Ref. [42]. Copyright 2022 John Wiley and Sons

In addition to the species of zinc salt, the concentration also determines the thermodynamic stability of electrolytes. For example, in ZnCl_2_-based electrolyte, with the increase in salt concentration, the potential window increases from 1.6 V versus SHE (5 M ZnCl_2_) to 2.3 V versus SHE (30 M ZnCl_2_) (Fig. 7a) [76]. Similarly, 3 M ZnSO_4_ possesses a higher OER potential than 1 M ZnSO_4_ (2.4 vs. 2.3 V) [62]. With the increase in zinc salt concentration, the presence of water clusters decreases or even disappears, and water molecules are transformed into the solvent sheath of Zn^2+^ cations. Importantly, the solvent sheath of Zn^2+^ ions is reconstructed by the anion (such as (Zn(H_2_O)_2_Cl_4_)^2−^ (ZnCl_4_)^2−^ replacing (Zn(H_2_O)_6_)^2+^) due to the ultra-deficient water molecules in high-concentration electrolyte [62, 91]. Thus, increasing salt concentration can reinforce the strength of O−H bond in water molecules. Moreover, adding another kind of soluble salt into electrolytes can also break hydrogen bond network of water clusters. For example, Zn^2+^ solvation sheath will gradually be changed with the increase in soluble LiTFSI in 1mZn(TFSI)_2_ electrolyte [39]. As shown in Fig. 7b, in the dilute electrolyte (1 M Zn(TFSI)_2_ + 5 M LiTFSI), Zn^2+^ are coordinated with six water molecules. At the 1 M Zn(TFSI)_2_ + 10 M LiTFSI electrolyte, TFSI^−^ replaces the original water sheath. As the concentration of LiTFSI increases to 20 M (1 M Zn(TFSI)_2_ + 20 M LiTFSI), the six water molecules of Zn^2+^ solvation sheath are replaced by six TFSI^−^. Water clusters and Zn(H_2_O)_6_)^2+^ are significantly suppressed, and the strength of O−H bond in water molecules is enhanced, contributing to improving the thermodynamic stability of electrolyte and inhibiting the water-induced parasitic reactions. Therefore, in high-concentration electrolyte (1 m Zn(TFSI)_2_ + 20 m LiTFSI), the accumulation of independent water molecules on the electrode surface is completely avoided during the de-solvation process of solvated zinc ions. Along this line, WiSE and DES electrolytes have been reported, where H_2_O molecules are coordinated with ions. As a result, the number of water clusters decreases, rendering a high thermodynamic stability for electrolytes. FTIR and NMR were used to monitor the change of water configurations [39]. As shown in Fig. 7c, with the increase in LiTFSI concentration, the peak of hydrogen bonding of H_2_O (at 3414 cm^−1^) fades away, which indicates hydrogen bond network is destroyed and the thermodynamic stability of water molecules is enhanced. The ^17^O NMR results show a lower chemical shift and a narrower half-height signal (Fig. 7d), revealing that previously excess O atoms of water are directly occupied and consumed by the added Li^+^. This unique electrolyte enables a nearly 100% CE plating/stripping process, and the volatility of water is deeply restrained. Similar to this are 21 M LiTFSI + 1 M Zn(OTf)_2_ [83] and 17 M NaClO_4_ + 3 M Zn(OTf)_2_ [90].

The high costs of OTf-based and TFSI-based salts limit their application. Therefore, many efforts have been made to develop WiSE using inexpensive salts as raw materials, such as ZnCl_2_ [76], Zn(OAc)_2_ [77], Zn(ClO_4_)_2_ [92], and ZnSO_4_ [42]. For example, the development of WiSE containing a single ZnCl_2_ salt has significantly improved thermodynamic stability of water, and parasitic reactions have been significantly inhibited [76, 91, 93]. As shown in Fig. 7e, the H-bond structure of water is broken and water molecules tend to coordinate with ions because of the strong electrostatic force between ions and water molecules. With the change of salt concentration, many kinds of Zn^2+^ solvation configurations appear in the electrolyte, such as Zn(H_2_O)_2_Cl_4_^2–^, ZnCl^+^, and ZnCl_4_^2−^. In the high-concentration electrolyte (30 m ZnCl_2_), the passivation product on zinc anode is converted from Zn(OH)_2_ in the original electrolyte (5 m ZnCl_2_) to ZnO, indicating that no water molecules participate in the parasitic reactions [76].

A binary ZnCl_2_ -based water-in-salt electrolyte (WiSE) with LiCl additive mitigates hydrogen evolution by transforming water clusters into solvated water molecules [89]. The relationship between O−H bond strength and salt concentration can be obtained by femtosecond stimulated Raman spectroscopy (FSRS) (Fig. 7f). In FSRS, 30 M ZnCl_2_ -based WiSE only exhibits a peak (at 3454 cm^−1^) and the hydrogen bond vibrations peaks disappear (at 3230 and 3420 cm^−1^), revealing that eliminate the existence of water clusters (hydrogen bonds) and enhances the thermodynamic stability of water in 30 M ZnCl_2_ -based WiSE.

A ternary hydrated salt electrolyte (HSE), 0.5 g Na_2_SO_4_·10H_2_O + 0.5 g ZnSO_4_·7H_2_O + 0.2 g MnSO_4_·H_2_O, is proposed to circumvent the parasitic reactions occurring on the zinc anode and cathode. In this WiSE, an unsaturated hydration structure (Zn(H_2_O)_n_^2+^, n < 6) is triggered, which suppresses the presence of water cluster and elevate the thermodynamic stability of electrolyte. As reflected by the Raman spectrum results, SO_4_^2−^ enters the solvation sheath of Zn^2+^ (cation-OSO_3_^2–^ in Fig. 7g) and the water cluster in electrolyte is absent (H_2_O^–^HOH in Fig. 7h), further confirming no water cluster in HSE [42]. Multicomponent (binary and ternary) WiSE of 30 M KAc + 1 M ZnAc_2_ [77], ZnCl_2_ + Zn(OAc)_2_ [7], 8 M NaClO_4_ + 0.5 M Zn(ClO_4_)_2_ [92], 30 M KAc + 3 M LiAc + 3 M ZnAc_2_ [86] have been investigated_._

Another surer-lean water electrolyte is deep eutectic solvent (DES) electrolyte [56, 94, 95]. The difference between DES and WiSE is that DES not only contains soluble salts, but also contains polar molecules (such as acetamide, urea, and succinonitrile), which are bound by hydrogen bond with H_2_O and enter the solvation sheath of Zn^2+^. For instance, a LiTFSI/Zn(TFSI)_2_/urea/water (molar ratio 1:0.05:3.8:2) DES electrolyte presents a wide electrochemical window (from − 1.26 to 1.66 V vs. SHE), implying that the DES electrolyte has good thermodynamic stability. Therefore, the zinc corrosion and HER are suppressed and a high capacity retention rate of > 90% is achieved in Zn|LiMn_2_O_4_ battery. In another works, water are completely replaced by the polar molecules in the DES electrolytes (Zn(TFSI)_2_/acetamide; ZnClO_4_·6H_2_O/succinonitrile; LiTFSI/Zn triflate/acetamide) to mitigate the parasitic reactions caused by aqueous solvent [56, 94, 96]. Although these electrolytes have achieved good electrochemical performance, this review focuses on AZIBs, so anhydrous electrolytes are not in the scope of discussion.

Undeniably, adequate ion conductivity is necessary to guarantee excellent electrochemical energy performances. The ion conductivity (σ) is expressed as follows [97]:

12 $$\sigma = \Sigma Z_{{\text{i}}} {\text{Fc}}_{{\text{i}}} \upmu_{{\text{i}}}$$

where the charge (*Z*_*i*_), concentration (*c*_*i*_), and mobility (*μ*_*i*_*)* of the ionic species.

In dilute electrolytes, the mobility (*μ*_*i*_) does not play a major role, and the conductivity of the electrolyte increases with increasing concentration (*c*_*i*_*)*. In the concentrating electrolyte, the ionic conductivity may be discounted due to the unfavorable mobility (high viscosity) of electrolyte [98]. Thus, the poor ion conductivity causes uneven ion deposition. When designing high-concentration electrolytes, it is necessary to comprehensively balance factors such as ion conductivity, solute type and concentration, and the stability of electrolyte.

Combining relevant work on organic lithium battery electrolytes, to keep the characteristics of contact ion pairs and cation–anion aggregate while reducing its viscosity and increasing ion conductivity, localized and interface high-concentrated electrolytes were proposed. A key characteristic of the localized high-concentration electrolytes (LHCE) is its solvation structure: salt-solvent clusters are distributed in the diluent molecules [99]. Therefore, LHCE has three major components: salt, solvating solvent, and non-solvating diluent. Nearly all the solvating-solvent molecules must be coordinated to the cation of the salt, so that little or no free water molecules remains in the electrolyte. A large fraction of anions is jointly coordinated with zinc ions, forming contact ion pairs (CIPs) and cation–anion aggregate. The diluent must offer little or no solubility to the salt and the diluent should preserve the high-concentration salt-solvent clusters in the formulated electrolyte. Nearly all the solvating-solvent molecules must be coordinated to the cation of the salt, so that little or no free water molecules remains in the electrolyte. Due to (1) the good solubility and high dielectric constant of water, (2) the larger bulk charge density of zinc ions, (3) the insolubility of zinc salts, and (4) few diluents that meet the above requirements, the LHCE of AZIBs has not been reported yet. Therefore, further exploration is needed for LHCE that can be applied in AZIBs. As for the implementation of interface high-concentrated electrolytes, zinc ions are concentrated at the electrode interface through functional artificial interfaces or structured electrodes, achieving the properties of high-concentration electrolytes. For example, a metal–organic framework (MOF) layer was constructed as front surface to maintain a super-saturated electrolyte layer on the Zn anode [48]. A Zn metal anode with accurately controlled nanopore structure is designed, in which the space charge distribution could be regulated and interface-localized concentrated electrolyte was enabled [100]. The above enhances the thermodynamic stability of the electrolyte in the surrounding environment of zinc electrode, thereby avoiding the occurrence of side reactions.

#### Additives Engineering for Enhancing Electrolyte Stability

In addition to increasing the salt concentration and selecting the appropriate zinc salt, additives can also be added to electrolytes to improve the thermodynamic stability of electrolyte. We classify the reported additives into three categories according to their distribution positions in electrolytes: (1) reducing the content of water clusters by destroying the structure of water clusters; (2) coordinating with cations and replacing the solvated water molecules of cations shell; and (3) promoting anions to enter cations shell and realizing that anions and additives co-coordinate the cationic solvent shell.

For the first type of additives, their mechanism and role are similar to that of increasing salt concentration in a dilute electrolyte, that is, additives break the original hydrogen bond network (water cluster) to convert water molecules into cationic solvent sheaths or fix water molecules through chemical bonds. The water molecules in Zn(H_2_O)_6_^2+^ will be further captured by additives to form unsaturated solvated structure (Zn(H_2_O)_n_^2+^, n < 6). This type of additive reported includes soluble salts (such as LiCl [88], Na_2_SO_4,_ and MnSO_4_ [42]) and organic substances containing polar functional groups to bind with water molecules, strengthening the O−H bonds in water molecules [103]. For example, LiTFSI [39] and LiCl [88], as nonelectrolyte salts and additives, widen the electrolyte stability window through the interaction between additives and water. Similarly, poly(ethylene glycol) (PEG) can widen the electrolyte stability window to 3.2 V due to the inductive donating effects of alkyl groups in PEG [103]. Furthermore, during the operation of AZIBs, a non-negligible role is that independent water molecules from the de-solvation process of Zn(H_2_O)_6_^2+^ at the electrode interface can be quickly and firmly fixed by additives.

**Fig. 8:**
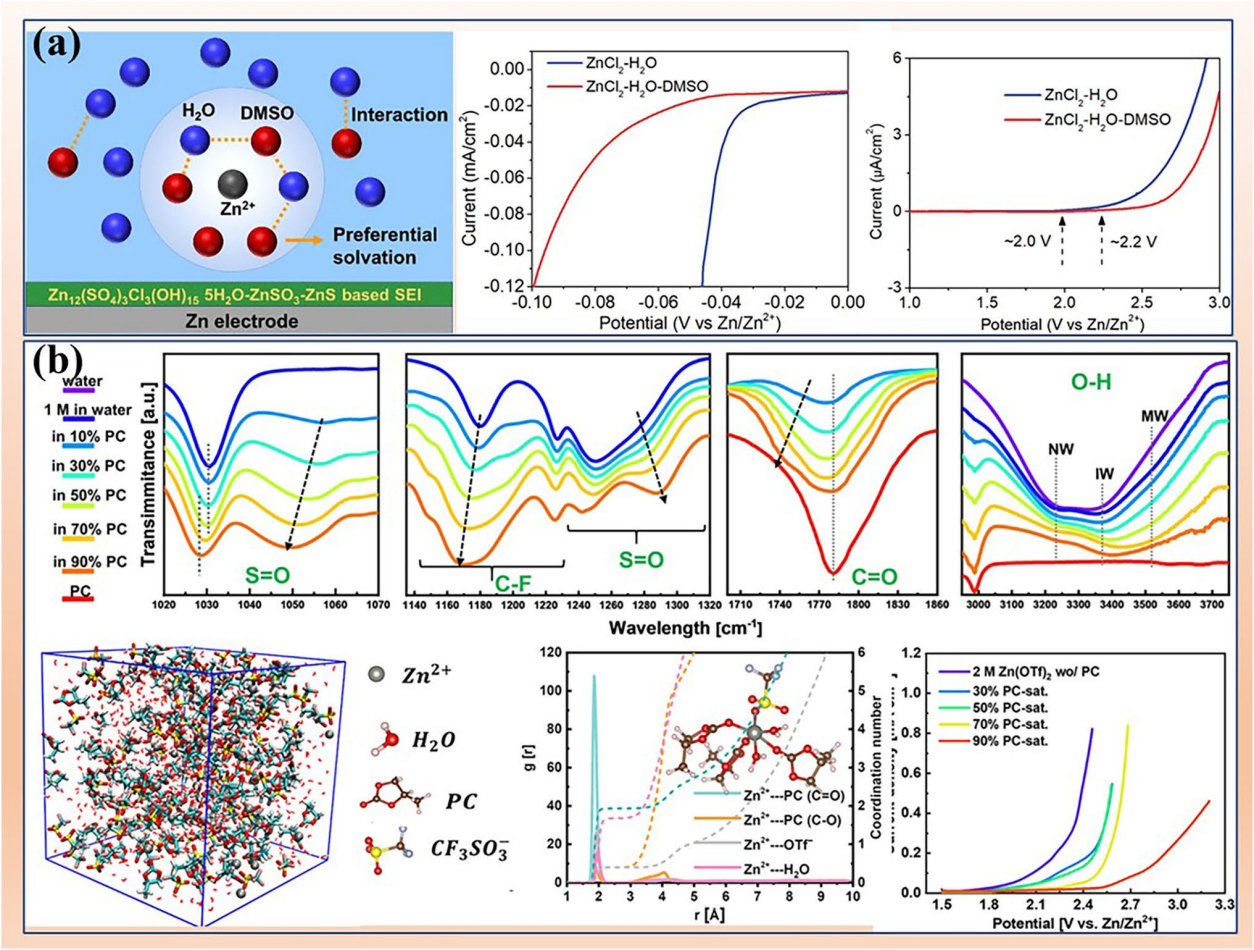
Additives improving electrolyte stability. **a** DMSO replacing H_2_O molecules in the cation’s solvation sheath and enhancing the stability of electrolyte. Reprinted with permission from Ref. [101]. Copyright 2020 American Chemical Society. **b** FTIR spectra of 1 M Zn(OTf)_2_ in pure water, PC, and mixing solvents with various ratios of water and PC; MD simulations of the Zn^2+^-solvation structures of 1 M Zn(OTf)_2_ in 50% PC; LSV curves of asymmetric Zn-Ti cells. Reprinted with permission from Ref. [102]. Copyright 2022 American Chemical Society

Another type of additives not only break the water cluster structure, but also replace original water sheath and coordinate with Zn^2+^ to become the solvent sheath [104]. For example, dimethyl sulfoxide (DMSO), with a higher Gutmann donor number (29.8) than water (18), exhibits a stronger tendency to coordinate with Zn^2+^ than water (Fig. 8a). Therefore, DMSO replaces H_2_O molecules in the Zn^2+^ solvent sheath [101]. Moreover, DMSO can also strongly break the hydrogen bond network of water cluster and then, interact with the H_2_O molecules, which is similar to the roles of the first type of additives mentioned above. Therefore, the electrolyte with DMSO shows a wider stability window than that without DMSO. In another work, glucose additive can enter solvation sheath of Zn^2+^ to from the configuration of glucose-Zn^2+^-5H_2_O [47]. Glucose additives effectively inhibit side reactions by reducing the content of water clusters and attracting independent water molecules, thereby enhancing the stability of electrolyte [105].

Yet another type of additives not only has the functions similar to the second additives, but also can promote the coordination of anions (such as TFSI^−^ and CF_3_SO_3_^−^) with cations, realizing the co-coordination structure of additives and anions with cations. For example, a co-solvent electrolyte based on the salting-in-effect is achieved by mixing of propylene carbonate (PC) and water [102]. In this electrolyte, the presence of PC can effectively regulate the Zn^2+^ solvation sheath, achieving a co-coordination solvation structure of PC and triflate anions with Zn^2+^ (Fig. 8b). As confirmed by FT-IR spectroscopic, PC can regulate the coordination environment of OTf^−^ anions, and coordinate with Zn^2+^ ions, leading the formation of [PC−OTf^−^−H_2_O] species. Moreover, the change of O−H stretching bands implies concentration of water cluster (network water) is reduced and the strength of the O−H bond in water molecules is enhanced. The MD simulations also prove the co-coordination solvation structure of PC and triflate anions with Zn^2+^.

Similarly, methanol and 1,2-dimethoxyethane (DME) were introduced into dilute electrolytes and to interrupt the Zn^2+^−H_2_O and H_2_O−H_2_O interactions to create unique Zn^2+^ solvation structure. The strengthened O−H stretching bands lead to a high Coulombic efficiency of Zn metal anode and wide potential window. In addition, the additive molecules and anions in the solvation sheath will decompose on the electrode surface to form protective layers (AEI), which isolates the electrolyte solvent and further effectively protects the electrode. This part will be summarized as the kinetic inhibition strategy of parasitic reactions (Sect. 4).

Compared with electrodes modification, electrolyte engineering offers a more multifunctional and comprehensive approach. It can not only improve the thermodynamic stability of electrolytes and electrodes, but also dynamically regulate the parasitic reactions in AZIBs. It needs to be emphasized that it is crucial the potential toxicity and pollution associated with the decomposition products of additives, which cannot be ignored. Therefore, we still need to carefully consider and conduct extensive experimental research when selecting additives.

### Strategies for Enhancing Zinc Anode Thermodynamic Stability

Thermodynamic stability of zinc anode is influenced by the intrinsic properties (e.g., internal energy, enthalpy, entropy, and Gibbs free energy). Zn metal has a hexagonal close-packed (HCP) crystal configuration, in which the lattice parameters are *a* = *b* = 2.63 Å; *c* = 5.21 Å. Among the crystal planes, Zn(002) facet as the densest packing crystal plane shows the lowest surface energy (2.08 eV nm^−1^), while the Zn(100) plane has the highest surface energy (4.60 eV nm^−2^). Therefore, adjusting electrode exposed texture is an effective strategy to improve the thermodynamic stability of Zn anode.

**Fig. 9:**
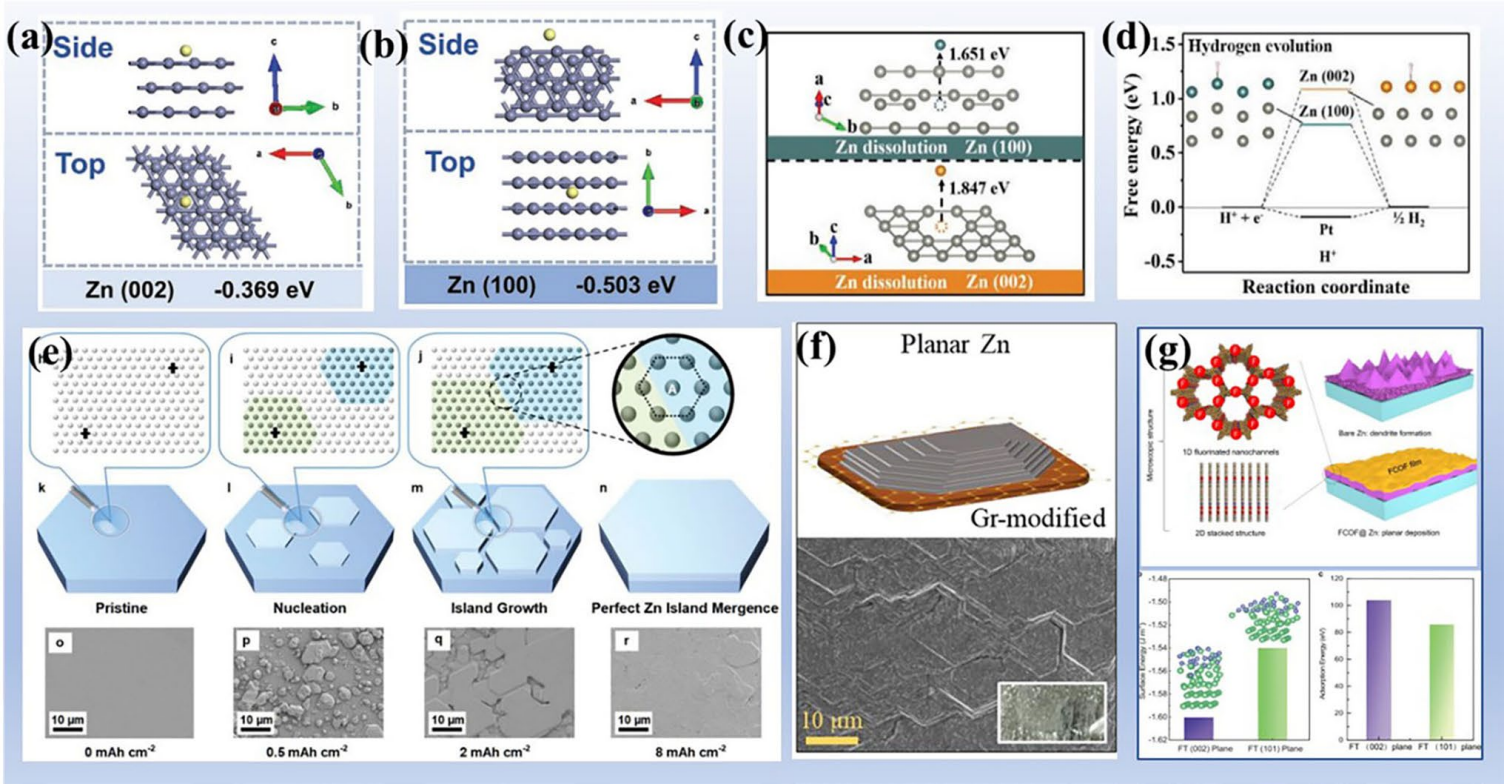
Zinc electrode stability and strategies for enhancing zinc anode thermodynamic stability. Adsorption energy of Zn atom on **a** Zn(002) and **b** Zn(100) crystal planes. Reprinted with permission from Ref. [106]. Copyright 2021 John Wiley and Sons. **c** Energy comparison of stripping zinc atom from Zn(002) and Zn(100) crystal planes. **d** Free energy of H adsorption on Zn(002) and Zn(100) crystal planes. Reprinted with permission from Ref. [108]. Copyright 2021 John Wiley and Sons. **e** Schematic diagram of electroplating zinc on single-crystal Zn substrate and the morphological evolution of electroplated Zn 2 mA cm^−2^ plating rate. Reprinted with permission from Ref. [111]. Copyright 2021 John Wiley and Sons. **f** Graphene (Gr) as heteroepitaxial substrate to deliver a highly planar Zn deposition. Reprinted with permission from Ref. [113]. Copyright 2019 American Chemical Society. **g** Fluorinated two-dimensional porous covalent organic framework (FCOF) film design and mechanism and the surface energy and adsorption energy of Zn (002) and Zn (101) crystal planes terminated by F atoms. Reprinted from Ref. [119] with permission from Springer Nature, Copyright 2021

The deposition/dissolution of zinc atom is closely related to the exposed facet. The adsorption energies of zinc atoms on Zn(002) and Zn(100) show that Zn(100) plane reveals a more negative adsorption energy(− 0.503 eV) for zinc atoms than that of Zn(002) (− 0.369 eV), implying zinc is preferentially deposited on the Zn(100) crystal plane(Fig. 9a, b) [106]. However, a higher stripping energy for zinc atom from Zn (002) (1.847 eV) than that of Zn(100) (1.651 eV) is delivered at the dissolution process (discharging state) (Fig. 9c), indicating a slow stripping rate along Zn(002) to block the homogeneous electrochemical dissolution of Zn anode. That is to say, more Zn(100) planes will be exposed during battery operation. The high surface energy of the exposed Zn(100) planes increases the reactivity of the zinc electrode and, more importantly, expands the contact area with the electrolyte, leading to the occurrence of significant parasitic reactions. In short, the exposed Zn(100) make zinc electrode more thermodynamic unstable [107].

In addition, the corrosion resistance of zinc metal is closely related to the exposed texture. The free energies of H adsorption on different Zn planes were calculated. As shown in Fig. 9d, the free energy of reference Pt(111) is − 0.09 eV, implying a fast HER process. The free energy of hydrogen adsorption on Zn(002) is larger than that of Zn(100) (1.0855 eV vs 0.7605 eV), indicating that HER can be suppressed by exposing more Zn(002) planes [108]. The higher corrosion resistance of Zn(002) has been confirmed by comparison with Zn(101) and Zn(103) in other works [109, 110]. In short, based on Zn(002) with high atomic binding energy and low interface energy, Zn anode with highly exposed Zn(002) plane shows high thermodynamic stability to resist parasitic reactions.

#### Texturing Zinc Electrode

To obtain highly exposed Zn(002) crystal planes, several strategies have been explored. One strategy is to directly use single crystal or highly textured zinc with exposed Zn(002) as anode for AZIBs [108, 111]. As shown in Fig. 9e, a single-crystal Zn-metal substrate provides nondifferential and stress-free deposition template along Zn(002) throughout plating process [111]. The stress-free, homoepitaxial deposition ensures the lowest energy barrier, the lowest internal stress, the smallest lattice distortion for the deposition layer, ensuring the minimization of internal energy. Moreover, due to the low-surface-energy facet favoring lateral growth and perfect lattice matching, the perfect inter-atomic splicing is maintained to ensure that no defects are formed along the grain boundary, thus eliminating the source of nonplanar Zn nucleation (Zn dendrites). Similarly, a preferentially exposed Zn(002) plane was produced by repeated rolling deformation, exhibiting no-dendrites and no-byproducts on surface. Highly stable and reversible zinc stripping/plating more than 500 h and CE of 97.71% are achieved [108]. However, the ultra-high price of single-crystal zinc and imperfect pre-treatment process for commercial zinc foil require researchers to continuously explore the construction of texturing zinc electrode.

#### Designing Heteroepitaxial Current Collectors for Zinc Deposition

Another strategy for regulating zinc texture is heteroepitaxial growth on a substrate owning a low lattice mismatch rate with Zn(002) (< 25%) [112]. To maintain small the interface energy and lattice distortion of electrodeposited layer, the Zn atoms tend to grow along substrate lattice to form semi-coherent (or coherent) interfaces. Note that heteroepitaxial growth is only related to the two-dimensional lattices of the exposed substrate and the crystal surface to be grown. As shown in Fig. 9f, a monolayer graphene (Gr) substrate as the heteroepitaxial substrate for zinc deposition to achieve a highly exposed Zn(002) anode and improved cycling performance [113]. Experimental observations and calculations demonstrate that the low lattice mismatch (7.5%) between the Gr layer and Zn(002) promotes the exposure of Zn(002) planes and results in a low interfacial energy (0.212 J m^−2^), thereby enhancing the thermodynamic stability of the electrodeposited layer and effectively suppressing parasitic reactions. In another work, based the compatibility between graphene and Zn, epitaxial Zn anodes achieve a high CE of above 99.7% in full cells and of 99.9% at current densities of 40 mA cm^−2^ in Zn|Cu battery, while conventional technologies can rarely realize [112, 114]. Inspired by the strategy of stabilizing zinc anode by heteroepitaxial growth, many epitaxial substrates have been developed, such as Sn [107], nitrogen (N)-doped graphene oxide (NGO) [115], Ag-Zn alloy [116], InGaZn_6_O_9_ [117], and MXenes [118].

#### *Regulating Zinc Deposition Texture *via* SEI and Working Current*

Yet another strategy is enhancing adsorption energy and reducing the diffusion energy barrier of Zn atoms along Zn(002) through SEI and working current. As mentioned above, Zn atoms have a smaller adsorption energy on the Zn(002) interface than that of Zn(100) (Fig. 9a, b), so Zn atoms are preferentially deposited on the Zn(100) to form dendrites. If this trend can be changed, the deposition of Zn atoms along Zn(002) plane can be effectively realized. For example, an ultrathin and porous fluorinated porous covalent organic framework (FCOF) film is developed as a protective layer for Zn anode (FCOF@Zn) to achieve the preferential growth of Zn(002) plane (Fig. 9g) [119]. In the FCOF membrane, electronegative F atoms not only interact strongly with zinc ions to promote the flow of zinc ions, but also lower the surface energy of Zn(002) relative to Zn(101), thereby enabling the deposition of zinc atoms along the Zn(002) plane and achieving a platelet morphology. Benefited from FCOF, Zn anodes show a life of more than 750 h at a density of 40 mA cm^−2^ and over 250 cycles in a Zn|MnO_2_ full cell with high areal capacity cathode. Based on this mechanism, numerous SEIs have been made to achieve favorable adsorption of Zn atoms on Zn(002) and deposition of zinc along Zn(002), such as rich-cyano SEI [120], zinc hydroxide sulfate [121], and poly(vinylidene fluoride-trifluoroethylene (P(VDF-TrFE)) [122].

The working current influences the overpotential of zinc deposition, which in turn affects the thermodynamic behavior of zinc ion deposition. As demonstrated, as the working current density increased from 1 to 100 mA cm^−2^, a transition in electroplating behavior was observed from a thermodynamically favorable Zn plate to a dynamically controlled packed crystal, and then to a diffusion limited metal pillar [66]. To circumvent the strategy of obtaining textured electrodes through epitaxial deposition, a new strategy of controlling the working current was used to obtain textured zinc electrodes [123]. It can be inferred the following conclusions. (i) At low current density (RCD < 0.1, RCD = i/i_L_, working current density/limiting current density), the epitaxial nucleation dominates, and the orientation of crystal nuclei strongly depends on the substrate, leading to the evolution of zinc electrodeposition on textureless substrates into randomly oriented zinc. (ii) At medium to high current densities (0.1 < RCD < 1), the increase in overpotential promotes the formation of horizontal Zn(002) nuclei, which weakens the influence of the substrate. Even on randomly oriented substrates, dense textured Zn(002) can also be formed. Based on these understandings, the preparation of textured Zn(002) electrode can be effectively achieved at medium current density.

Highly exposed Zn(002) anodes can be achieved by some novel electrolytes [124, 125], additives [126, 127], and separator [128, 129], regulating regulate the thermodynamic behavior of Zn deposition [130]. The common goal of these strategies is to control the atomic-scale deposition of Zn ions, leading to the exposure of the Zn(002) crystal plane. Therefore, these works provide meaningful reference for further construction of zinc anode without parasitic reaction.

There are various approaches to modify the properties of a zinc anode, including 3D structuring, alloying, and modifying the Zn lattice through processes like doping and creating vacancies. The 3D Zn anode expands the surface energy and increases the internal energy, thus reducing the thermodynamic stability [131–133]. Alloying of zinc anode leads to crystal lattice distortion, which increases the internal energy of anode, and can also lead to micro-battery corrosion [134]. As for the modification of Zn lattice, crystal lattice distortion caused by doping and vacancy increases the internal energy. Therefore, from the perspective of electrode thermodynamics, the 3D structure anode, alloying anode, and the modification of Zn lattice are unreasonable. Although they can effectively inhibit zinc dendrites [135–137], serious parasitic reactions may be promoted.

### Decoupling Battery Enhancing Zinc Anode and Electrolyte Stability

The thermodynamic stabilities of electrolytes and electrodes are sensitive to the pH environment. As shown in Fig. 3a, an alkaline environment is more resistant to the electrochemical HER at anode side, where a high overpotential for HER is exhibited. Moreover, the Zn anode in alkaline environment shows a more negative electrode potential than that in acidic environment, delivering a higher output voltage. Conversely, acidic environment inhibits the anodic process of water molecules (OER) at cathodes side. It should be noted that an acidic environment allows for the intercalation of H^+^ ions into cathodes and inhibits the formation of passivation products. Therefore, creating a decoupled electrolyte environment for anodes and cathodes can enhance the thermodynamic stability of both electrolytes and electrodes, ultimately expanding the electrochemical window.

**Fig. 10:**
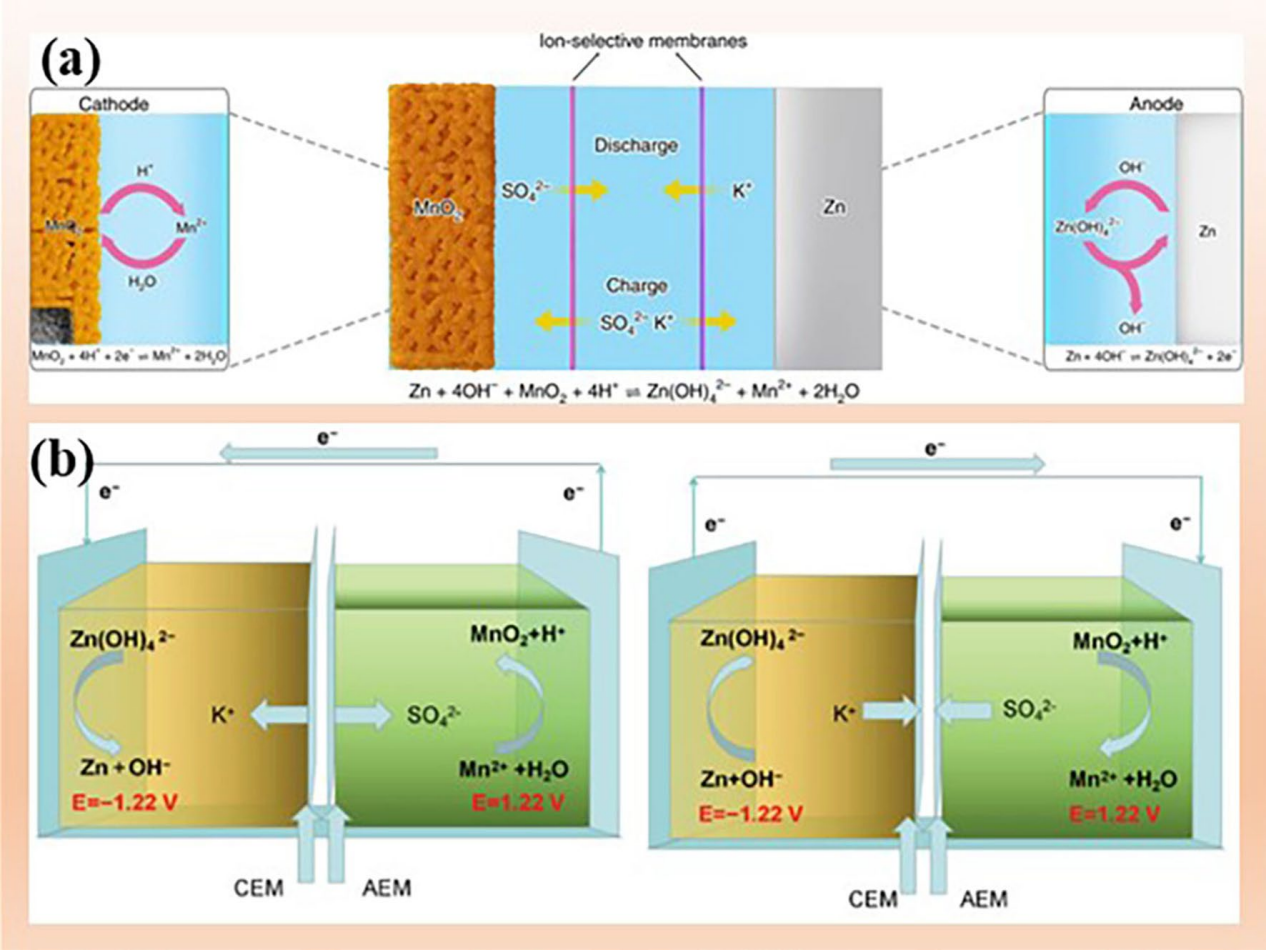
Decoupling electrolytes to enhance the stability of electrolytes and electrodes. **a** Schematic illustration of the cell structure and chemical reactions of a three-chamber Zn|MnO_2_ system. Reprinted with permission from Ref. [138]. Copyright provided by Springer Nature. **b** Decoupling electrolytes with a bipolar membrane (BPM). Schematic illustration of mechanism of Zn|MnO_2_ battery at charging state and discharging state. Reprinted with permission from Ref. [139]. Copyright 2020 John Wiley and Sons

The traditional two-chamber battery with a transparent separator (such as glass fiber and filter paper) cannot effectively isolate the electrolyte. With the development of membrane materials, a three chambers battery with two ion selective membranes can effectively decouple the electrolyte environment. For instance, an aqueous three-chamber Zn|MnO_2_ cell was reported, where the Zn anode with alkaline electrolyte (6 M KOH + 0.2 M ZnO + 5 mM vanillin) and MnO_2_ cathode with acidic catholyte (3 M H_2_SO_4_ + 0.1 M MnSO_4_) are separated by a sandwiched chamber filled with neutral electrolyte (0.1 M K_2_SO_4_) and two ion-selective membranes (Fig. 10a) [138]. The anode delivers a low potential of − 1.199 V versus SHE by the reaction of Zn/Zn(OH)_4_^2−^; while the MnO_2_ offers a voltage of 1.2 V versus SHE based on the dissolution/deposition of MnO_2_/Mn^2+^. The decoupled system of Zn|MnO_2_ exhibits a high open-circuit potential of 2.83 V and almost constant discharge capacity at various current densities. Significantly, after 116 deep cycles (200 h), only 2% capacity decay could be achieved, further mitigating parasitic reactions in AZIBs (Table 1).

**Table 1:** Comparison of electrochemical performance

Zinc salts	Concentration	Operation window (OW) (V) or electrochemical window (EW) (V) (vs Zn/Zn^2+^)		References
ZnSO_4_	1 M	OW:0.2 ~ 1.3	EW: 2.3	[1, 51, 62]
	3 M	OW:0.8 ~ 1.9	EW:2.4	[51, 81]
Zn(ClO_4_)_2_	1 m	OW:0.2 ~ 1.4	EW:2.4	[73, 74]
Zn(CF_3_SO_3_)_2_	1 M	OW:0.2 ~ 1.5	EW:1.98	[51, 82, 83]
	3 M	OW:0.8 ~ 2	2.5	[51, 62]
Zn(CH_3_COO)_2_	1 M	/	EW: -1.0 to 1.95 (vs. Ag/AgCl)	[77]
Zn(OTf)_2_	4 M	0.2 ~ 1.9 CE:98%	EW:2.0	[84]
1 m Zn(TFSI)_2_ + 20 m LiTFSI		0.8 ~ 2.1 CE:100%	/	[39]
1 M Zn(CF_3_SO_3_)_2_ + 21 M LiTFSI		CE:100% OW:0.9 ~ 2.1	EW:2.6	[83]
0.4 M Zn(CF_3_SO_3_)_2_ + 8 M NaClO_4_		OW:0.8 ~ 1.9	EW:2.4	[85]
31 m KAc + 1 m ZnAc_2_		OW:0.8 ~ 2.0 CE:99%	EW:-1.45 to 1.95 (vs. Ag/AgCl)	[77]
30 m KAc + 3 m LiAc + 3 m ZnAc_2_		OW:1.5 ~ 2.1 CE:99.6%	EW:2.25	[86]
ZnCl_2_ + Zn(OAc)_2_·2H_2_O (molar ratio 10:6)		OW:0.7 ~ 1.5 CE:99.59%	EW:-0.8 to 3 (vs. Ag/AgCl)	[87]
15 m ZnCl2 + 1 m LiCl		OW:0.6 ~ 1.6 V CE:98%	EW:2.4	[88]
30 m ZnCl_2_ + 5 m LiCl		OW:1.55 ~ 1.95 V CE: 99.7%;	/	[89]
17 m NaClO_4_ + 3 m Zn(OTf)_2_		OW:0.8 ~ 2.1 V; CE: 99.96%;	EW = 1.8 (vs Ag/AgCl)	[90]
1 M Zn(OTf)_2_ + 21 M LiTFSI		OW:0.8 ~ 2.1 CE = 100%	EW:2.6	[83]
ZnCl_2_·2.33H_2_O		Zinc–Air Battery; Discharge platform≈1.2; CE:98.7%	EW:2	[91]
0.5gNa_2_SO_4_·10H_2_O + 0.5 g ZnSO_4_·7H_2_O + 0.2 g MnSO_4_·H_2_O		OW:1 ~ 2.1	EW:2.55	[42]

Similarly, a bipolar membrane (BPM), replacing the sandwiched chamber in Fig. 10b, is applied to separate the alkaline electrolyte and acid electrolyte for a Zn|MnO_2_ battery with dual dissolution/deposition mechanism to deliver a high voltage of 2.44 V and ultrahigh specific energy density of 1503 Wh kg^−1^ (Fig. 10b), which is higher than the traditional batteries [139].

This achievement is made possible by utilizing functional membrane materials like Nafion NR212 [140] and Li_1+x+y_Al_x_Ti_2x_Si_y_P_3y_O_12_ [141], which serve to isolate the electrolyte and facilitate ion transfer. Compared to the traditional battery with a permeable separator, such a design improves the thermodynamic stability of AZIBs, mitigates parasitic responses, and widens electrolyte working window [140–145]. This strategy should be applicable for other water-based energy storage systems to improve the thermodynamic stability of electrolytes and electrodes, and heighten output voltages and capacities (Table 2) [140, 144]. Besides, the ion-selective membranes showing low ionic conductivity, high price, and poor mechanical strength discount the advantages of decoupling battery.

**Table 2:** The performance of decoupling systems of reported Zn-based batteries

Anode/Cathode	Battery configuration	Electrochemical performance	References
Zn/MnO_2_	Three chambers separated by ion exchange membranes; Zn (6 M KOH + 0.2 M ZnO + 5 mM vanillin)|(0.1 M K_2_SO_4_)|MnO_2_ (3 M H_2_SO_4_ + 0.1 M MnSO_4_)	Open-circuit voltage:2.83 V; Energy density: 1621.7 Wh kg^−1^; Discharge plateau: 2.71 V; Specific capacity:616 mAh g^−1^	[138]
Zn/MnO_2_	Two chambers separated by a BPM; Zn (2.4 m KOH + 0.1 m Zn(CH_3_COO)_2_)|BPM| carbon cloth (0.5 m H_2_SO_4_ + 1.0 m MnSO_4_)	Battery stability window: 3 V; Zn-MnO_2_ output voltage: 2.44 V; Energy density: 1503 Wh kg^−1^	[139]
Zn/MnO_2_	Two chambers separated by BPM; 3 M NaOH + 0.3 M ZnO|BPM|3 M MnSO_4_ + 0.3 M H_2_SO_4_ + 0.06 M NiSO_4_	Battery stability window:3.45 V; Discharge plateau:2.44 V; Energy density:650 Wh kg^−1^; Specific capacity:270 mAh g^−1^	[142]
Zn/Br_2_	Two chambers separated by BPM; 2 M KOH + 0.02 M Zn(CH_3_COO)_2_|BPM|1 M KBr + Br_2_ + 0.5 M H_2_SO_4_	Battery stability window:3.1 V; Discharge plateau:2.1 V; Specific capacity:395 mAh g^−1^	[143]
Zn/KMnO_4_	Two chambers separated by Li_1+x+y_Al_x_Ti_2x_Si_y_P_3y_O_12_ (LATSP); 1 M H_2_SO_4_|LATSP|2 M KOH + 2 M LiOH	Battery stability window: 3 V; Discharge plateau:2.8 V; Specific capacity:510 mAh g^−1^; Energy density:454 Wh kg^−1^	[141]

## Restraining Parasitic Reactions from the Perspective of Reaction Kinetics

In addition to regulating the inherent stability of battery modules in AZIBs system (Sect. 3), it can also be achieved by external means, such as reducing the active sites and building artificial AEI. In this section, we mainly reviewed advanced strategies for suppressing parasitic reactions from the perspective of kinetics.

Blocking the contact area between electrolyte and electrodes can reduce the side reactions. One approach is to utilize adsorbents that can be adsorbed on zinc active sites, preventing the contact between reaction precursors (such as Zn metal and H_2_O). In Sect. 4.1, we classify adsorbents into organic and inorganic categories based on their material properties. The other is the employment of electronically insulating but ionically conductive AEIs on zinc electrodes, including artificial SEI and derivative SEI (Sect. 4.2). It is known that the emergence of dendrites will enlarge the exposed area and promote the parasitic reactions [146, 147]. Therefore, adsorbents and AEIs are widely used to inhibit zinc dendrites.

### Shielding Active Site through Adsorbents

The adsorbents interact with the zinc substrate through electrostatic forces, functional groups, and other physical effects, without undergoing any reaction with the zinc electrode or decomposing themselves. Some adsorbents can adsorb on the electrode surface and react with zinc anode to derive SEI. This will be discussed in the derivative-SEI strategy for zinc anode (Sect. 4.2.1). Adsorbents need to possess thermal, chemical, and electrochemical stability, as well as strong adsorption capabilities to inhibit water contact with the electrodes. Both organic and inorganic adsorbents are added to electrolytes as additives.

#### Organic Adsorbents

**Fig. 11:**
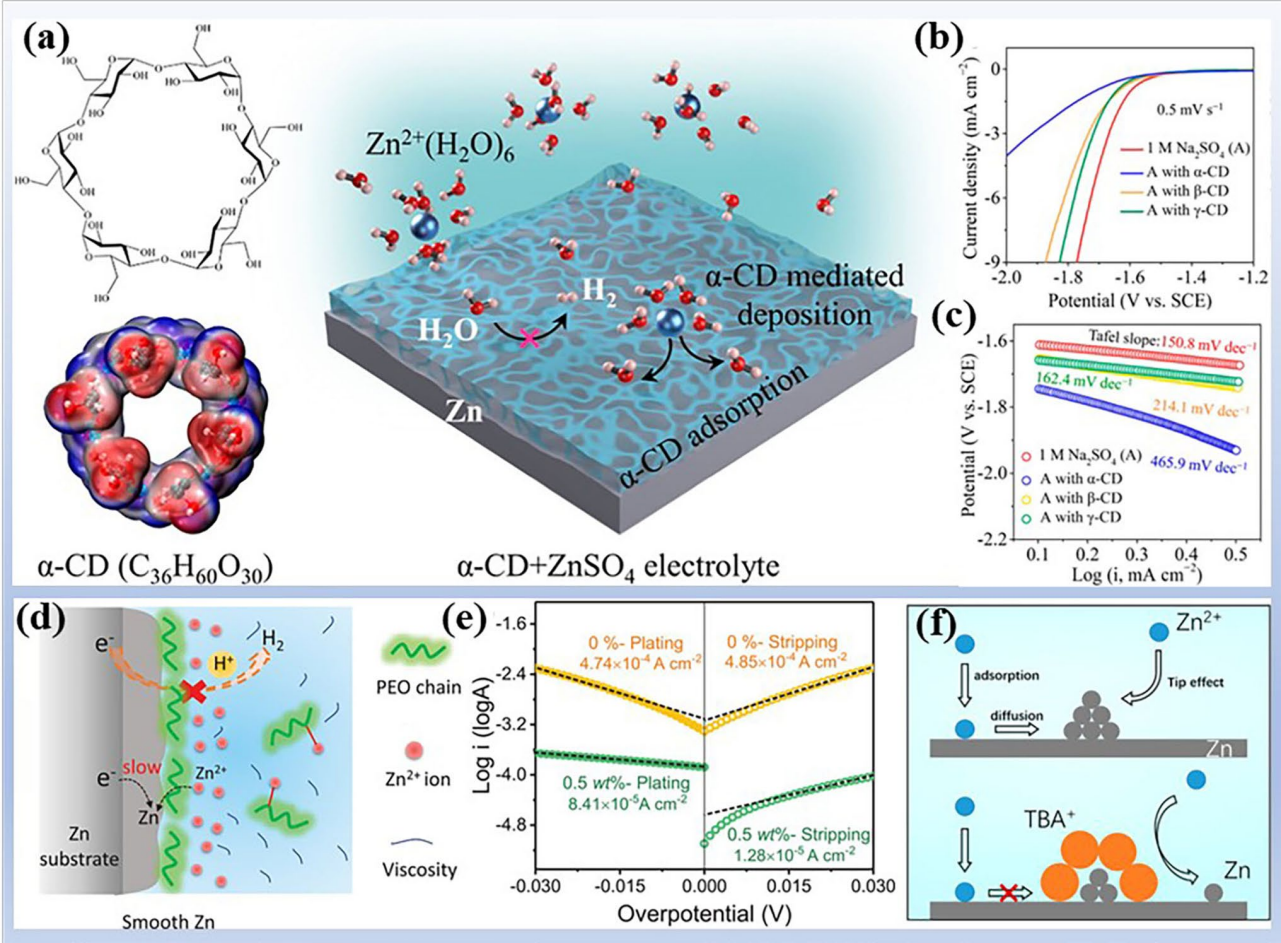
Organic adsorbent adjusting the Zn/electrolyte interface to avoid parasitic reactions. **a** Molecular structure and the electrostatic potential distribution of α-cyclodextrin (α-CD) in top view. Schematic illustration of Zn plating behaviors in 3 M ZnSO_4_ + 10 mM α-CD electrolyte. **b** and **c** Hydrogen evolution polarization curves at 0.5 mV s^−1^ and **e** corresponding Tafel plots of a Ti electrode in 1 M Na_2_SO_4_ solution with 10 mM α/β/γ-CD additives. Reprinted with permission from Ref. [150]. Copyright 2022 American Chemical Society. **d** Schematics of Zn deposition with PEO polymer adsorbent. **e** Tafel plots for Zn electrodes in 1 M ZnSO_4_ electrolyte with and without PEO polymer adsorbent. Reprinted with permission from Ref. [148]. Copyright 2020 John Wiley and Sons. **f** Schematics of the Zn^2+^ ion diffusion and reduction processes in 2 M ZnSO_4_ electrolyte without (upper part) and with (lower part) 0.05 mM TBA_2_SO_4_ adsorbent. Reprinted with permission from Ref. [152]. Copyright 2020 American Chemical Society

The application of organic adsorbents such as cetyltrimethylammonium bromide (CTAB), sodium dodecyl sulfate (SDS), polyethylene glycol (PEG), thiourea (TU), polyethylene oxide (PEO), polyacrylamide (PAM), and cyclodextrin (CD) in the electrolyte showed good barrier performance of parasitic reactions in AZIBs [126, 148–153]. For instance, adding 10 mM α-CD into 3 M ZnSO_4_ electrolyte restrains the parasitic reaction on Zn surface [150]. As illustrated in Fig. 11a, the outer surface of α-CD molecules shows a concentrated negative charge (blue area), which means that it has strong adsorption capacity on metal substrates. Authors proved the α-CD (− 0.87 eV) has a higher adsorption energy than β-CD (− 0.45 eV), γ-CD (− 0.21 eV) and H_2_O molecule, indicating a reduced H_2_O adsorption on zinc substrate in electrolyte containing α-CD additives. As indicated by the Tafel and LSV curves (Fig. 11b, c), the decreased cathodic current density and HER kinetics with CD additives follows an order of α-CD > β-CD > γ-CD, underscoring the strong adsorption of the α-CD. The self-adsorbing α-CD layer on Zn electrode as a protective layer reduces the contact frequency between H_2_O and the electrode interface, thus suppressing parasitic reactions. As a result, the α-CD additive contributes toward mitigating parasitic reactions and enabling uniform plating of Zn anodes.

Typically, some organic adsorbents (such as PEO and PAM) with rich polar functional groups can not only adsorb on zinc electrode interface to block the contact between the solvent and the electrode interface, but also enhance the concentration of zinc ion at the electrode interface through functional groups to promote zinc ion reaction [148, 149]. As shown in Fig. 11d, long-chain PEO adsorbent was introduced into aqueous electrolyte, which can be adsorbed on zinc surface to form PEO layer and improve the stability of zinc metal anodes. Moreover, the strong interaction between ether groups (−O−CH_2_) and Zn^2+^ enables the enrichment of Zn^2+^ at the electrode interface and achieves a uniform zinc plating/stripping process. Thus, the adsorption of PEO inhibits the occurrence of side reactions and promotes effective charge transfer (Zn^0^ ↔ Zn^2+^). A decreased exchange current density for self-corrosion of zinc evidenced the adsorption layer prevents the occurrence of parasitic reactions. As a result, the Zn metal anode can achieve a more than 3000 h lifetime at 1 mA cm^−2^/1 mAh cm^−2^ in 1 M ZnSO_4_ + 0.5 wt% PEO. PAM, a rich acyl group polymer, can be adsorbed on the Cu collector to prevent parasitic reactions [148]. Remarkably, the amount of PAM should be within the proper range to balance the coverage of adsorption layer and ionic conductivity. It is proven that PAM can prevent zinc corrosion. In addition, the acyl groups can adsorb Zn^2+^ along the PAM chains. In brief, this kind of organic adsorbents adsorbs on Zn surface reducing the contact frequency between water and Zn surface and adjust the Zn^2+^ flux.

Certain small molecule adsorbents with charge and polarity exhibit remarkable selectivity in adsorbing exposed sites [151, 152, 154]. It is well known that zinc dendrites protrude from the zinc substrate, which endow high electric field strength and high exposure area. Polar (charged) molecules can block the active sites (dendrites) through molecules adsorbed on the protrusions. As shown in Fig. 11f, cationic surfactant TBA_2_SO_4_ as additive was introduced aqueous electrolyte and the non-redox TBA^+^ ions are adsorbed on the Zn anode surface to regulate the initial nuclei formation and inhibit the active sites. When the non-redox TBA^+^ ions selectively adsorb on protrusions to form a TBA^+^ cation layer, Zn^2+^ ions are plated on the adjacent area of the active site until a smooth deposit is formed. Similar to the above organic cationic surfactant, the highly polarized diethyl ether molecules preferentially adsorb on tips of protrusions to decrease the exposed area of active zinc and greatly alleviate the occurrence of side effects [151].

The inhibition effect of surface organic adsorbent on parasitic reactions is related to chain length, concentration, functional groups, and electrolyte pH. The compactness of the adsorptive layers also significantly impacts cell performance, as greater compactness results in higher charge resistance. Therefore, many factors should be balanced when selecting an adsorbent.

#### Inorganic Adsorbents

**Fig. 12:**
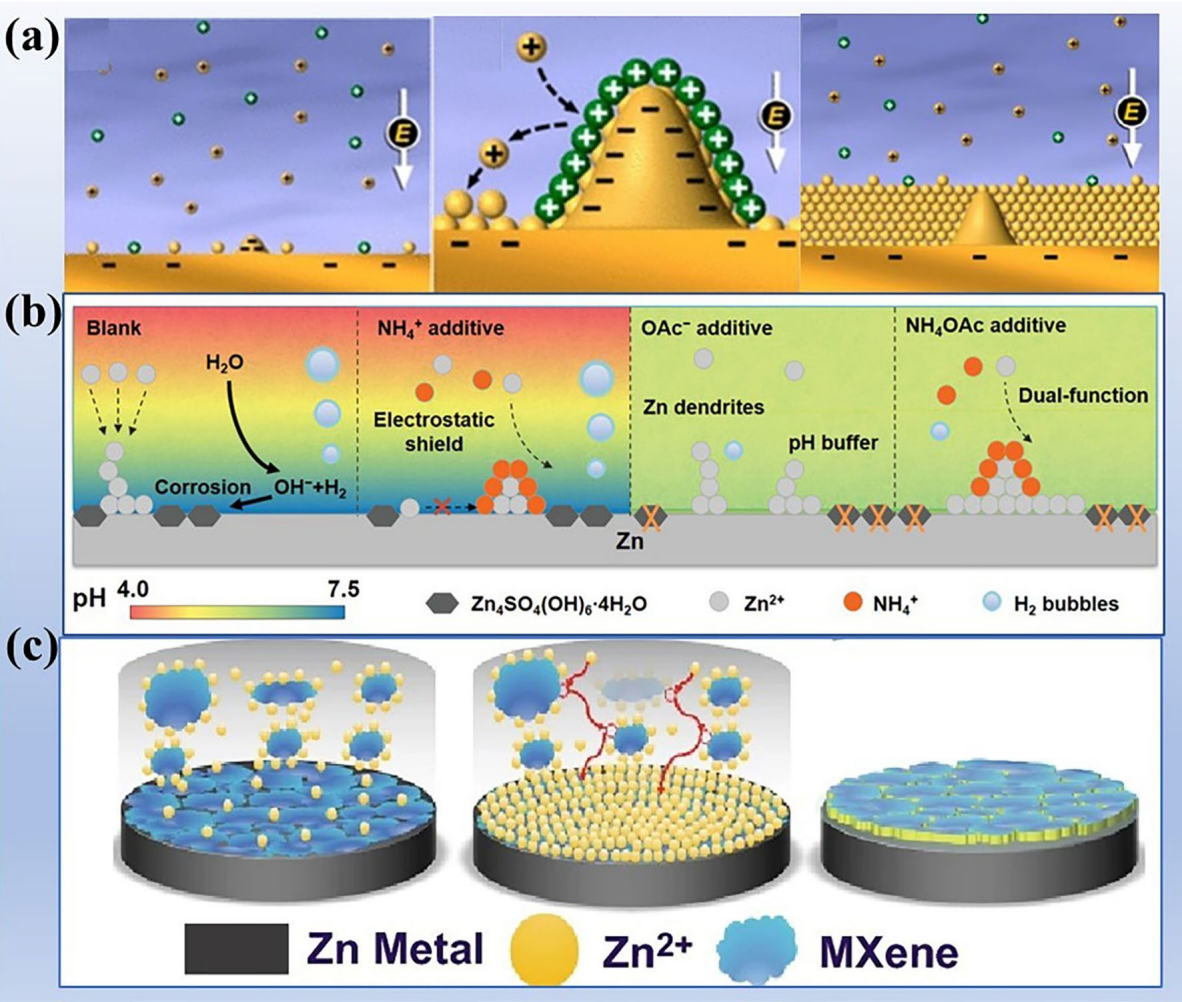
Inorganic adsorbents shielding active sites to inhibit parasitic reactions. **a** Illustration of the electrostatic shield mechanism. Reprinted with permission from Ref. [166]. Copyright 2013 American Chemical Society. **b** Schematic illustration of the roles of NH_4_^+^ and OAc^−^. Reprinted with permission from Ref. [165]. Copyright 2022 John Wiley and Sons. **c** Schematic illustration of the effect of MXene additive on the Zn deposition process. Reprinted with permission from Ref. [161]. Copyright 2021 Springer nature

Inorganic adsorbent includes soluble ionic adsorbent (such as LiCl [155], indium sulfate [127], boric acid [127], La(NO_3_)_3_ [156], ammonium hydroxide [157]) and insoluble adsorbent (ZnF_2_ [158], SnO_2_ [127], g–C_3_N_4_ [159], graphene quantum dots [160], Ti_3_C_2_T_x_ [161]). Soluble inorganic adsorbents primarily rely on the electrostatic shielding effect to limit the growth of active sites. Specifically, a cation additive, that has a lower reduction potential than that of Li^+^, can be adsorbed on protrusions but not consumed during Li deposition to eliminate active sites. As depicted in Fig. 12a, during the deposition process, the active sites exhibit a strong electrical field, so more cations (including Li^+^ and cation additive) will be adsorbed around the active sites. The adsorbed Li^+^ are reduced, while cation additives accumulate to repel the entry of Li^+^ ions, prompting further deposition of Li^+^ ions in adjacent regions of the active sites until a smooth deposition layer is formed. Based on the same mechanism, additives such as LiCl [155], Na_2_SO_4_ [162], CeCl_3_ [163], La(NO_3_)_3_ [156], and MnSO_4_ [164] have been reported.

With the exploration of soluble salt adsorbents, the role of anions is constantly recognized and understood. For example, ammonium acetate (NH_4_OAc) additive was introduced, in which NH^4+^ serves as electrostatic shielding adsorbent and OAc^−^ acts as pH buffer (Fig. 12b**)** [165]. The pH buffer regulates H^+^/OH^−^ concentration to endow fine pH value of ≈5.14, which significantly suppresses the side reactions. As a result, the Zn anode sustains a high CE of 99.7% due to the synergistic effect of the NH^4+^ and OAc^−^. Another example, LiCl additive ionizes out Li^+^ and Cl^−^ [165]. Cl^−^ facilitates the transport of Zn^2+^ at anode interface and Li^+^ serves as electrostatic shielding adsorbent to suppress the formation of active sites. As for the insoluble inorganic adsorbent, the action mechanism is the same as that of organic adsorbent, mainly including reducing the contact frequency of precursors of side reactions and regulating the flow of zinc ions. As illustrated in Fig. 12c**,** Ti_3_C_2_T_x_ as electrolyte additive can combine Zn^2+^ via electrostatic interaction and then, settle on the surface of Zn anode. The Ti_3_C_2_T_x_ layer can homogenize the dispersion and flux of Zn^2+^ in the deposition process and block the active sites. As a result, Zn metal anode delivers a nearly 100% CE with 2 M ZnSO_4_ + 0.05 mg mL^−1^ electrolyte.

Physical adsorption mechanisms have been extensively studied in the field of electrolytes to protect zinc anodes. However, the discontinuous distribution and weak binding force of these physical adsorbents on the electrode surface result in limited inhibition of side reactions. Moreover, this method increases the viscosity and reduces the ionic conductivity of the electrolyte. Therefore, protective layer as an effective solution has been widely studied in AZIBs. It can physically separate the electrolyte and the electrode while allowing the passage of Zn^2+^. In the following sections, we will review the latest advancements in this area.

### Constructing SEI for Conducting Zinc Ions

Unlike non-aqueous batteries, the absence of an in situ solid electrolyte interphase (SEI) on the Zn anode leads to persistent parasitic reactions. Taking inspiration from organic batteries, the use of derivative SEI and artificial SEI has been explored to mitigate these parasitic reactions. Both strategies have proven effective in suppressing parasitic responses and facilitating the deposition of zinc ions. In this section, we will review the recent advancements in SEI construction.

#### Derivative SEI

Derivative in situ SEI can be formed through spontaneous chemical reactions in the battery system, or the decomposition of additive and anions below the LUMO potential. The derived SEI exhibits a seamless connection with the zinc electrode and ensures a uniform distribution, effectively regulating active sites and inhibiting parasitic reactions.

**Fig. 13:**
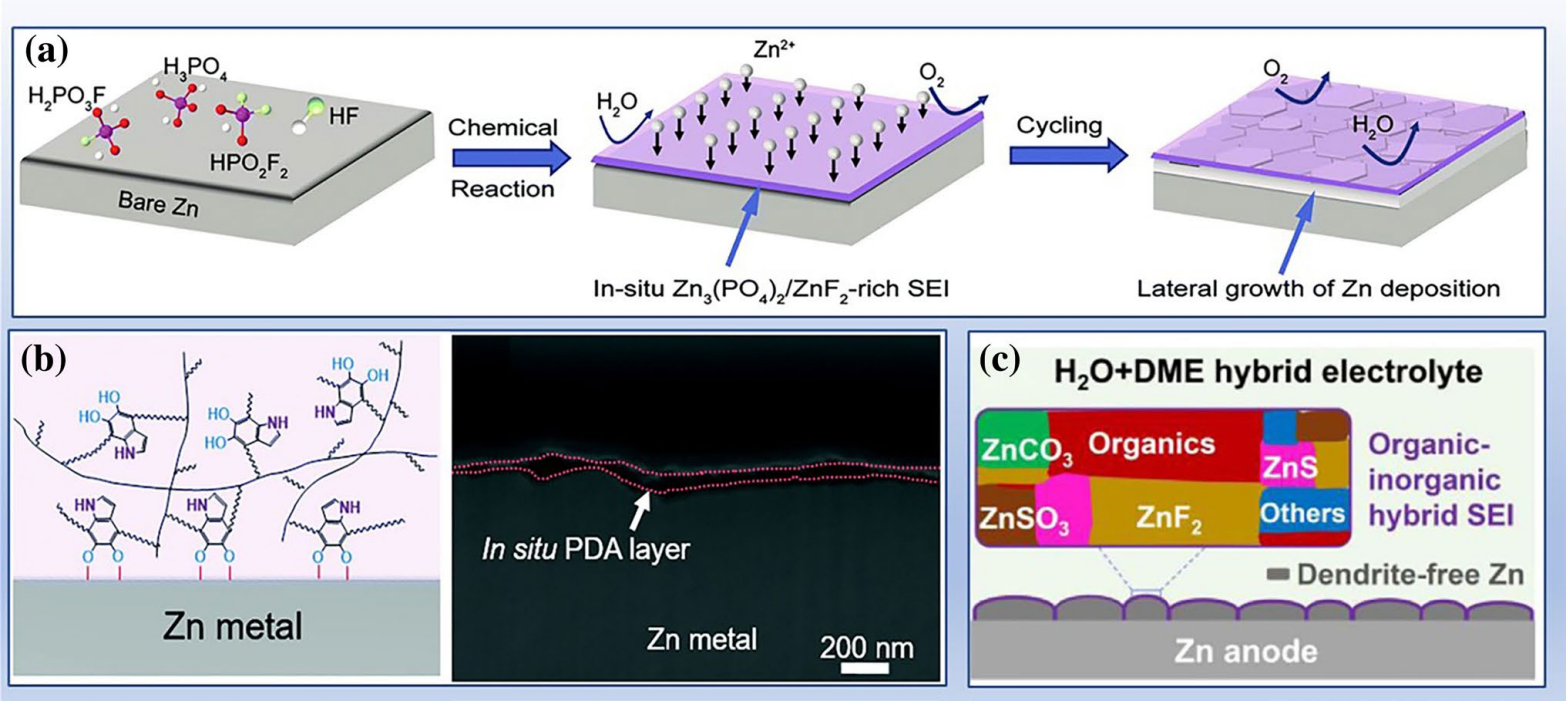
Strategies for derivative SEI to restrict parasitic reactions. **a** Schematic illustration of inorganic KPF_6_ additive to derive SEI. Reproduced with permission from Ref. [167]. Copyright 2021 Royal Society of Chemistry. **b** Schematic illustration of the in situ polydopamine (PDA) SEI and side-view focused ion beam SEM of the PDA SEI after 10 cycles. Reproduced with permission from Ref. [170]. Copyright 2021 Royal Society of Chemistry. **c** Schematic illustration of organic–inorganic SEI by the decomposition of DME and OTF^−^. Reproduced with permission from Ref. [171]. Copyright 2022 Elsevier

As an example of chemical reaction forming SEI, the thermodynamic unstable KPF_6_ in aqueous environment was decomposed to derive an in situ SEI (Fig. 13a) [167]. Specifically, PF_6_^−^ reacts with water to form acidic compounds such as POF_3_, HF, HPO_2_F_2_, H_2_PO_3_F, and H_3_PO_4_, which spontaneously react with Zn anode to form an in situ SEI containing Zn_3_(PO_4_)_2_ and ZnF_2_ on the Zn anode. Benefiting from the derivative SEI, the zinc anode exhibits a fast kinetics for Zn^2+^ transference and deposition and an enhanced anti-corrosion ability. Similarly, trace Zn(H_2_PO_4_)_2_ (0.025 M) [168], Zn(NO_3_)_2_ (20 mM) [169], and ZnF_2_ (0.08 M) [158] were introduced to electrolytes to derive dense and uniform SEI.

Certain additives can directly polymerize to derive SEI during battery operation process. For example, dopamine (DA) is a universal adsorbent, which can be converted into polydopamine (PDA) by autoxidation in air or electrochemical polymerization in aqueous solution [170]. As shown in Fig. 13b, an in situ PDA was constructed on Zn anode by introducing DA into electrolyte, which serves as an isolation layer to suppress side reactions. Meanwhile, the SEI layer regulates the homogeneous distribution of Zn^2+^ and facilitates uniform Zn nucleation. Therefore, Zn electrode with PAD SEI delivers a high average CE of 99.5% for over 1000 cycles.

Another derivative SEI is to adjust the solvation structure of Zn^2+^ and reduce anions of zinc salt and organic co-solvents. In this derivative SEI, the inorganic inner layer and the organic outer layer are usually included. For example, 1,2-dimethoxyethane (DME) can create a unique Zn^2+^ solvation structure with the co-participation of DME and OTF^−^, which derive an organic outer layer and an inorganic ZnF_2_ − ZnS inner layer on Zn anode by the decomposition of DME and OTF^−^** (**Fig. 13c**)** [171]. The SEI protects the Zn surface from water attack, guides the diffusion of Zn^2+^, and enables Zn anodes to achieve a high CE of 99.7% and a long cycling life of 5000 h. Similarly, dimethyl sulfoxide (DMSO) and acetamide (Ace) have been shown to modulate cation solvation and form organic/inorganic hybrid in situ SEI [101]. In another work, Ace and FSI^−^ anions co-ordinate with Zn^2+^ in the form of [Zn(TFSI)_m_(Ace)_n_]^(2−m)+^ (*m* = 1 ~ 2, *n* = 1 ~ 3) to promote the formation of a hybrid SEI with ZnF_2_ inner layer and organics outer layer (containing S and N), delivering a capacity maintenance of 92.8% over 800 cycles (99.9% CEs after activation) [94].

In summary, derived SEIs play a crucial role in isolating solvent from Zn anodes, effectively inhibiting parasitic reactions on the Zn surface. However, a notable disadvantage of these derived SEIs is the rapid consumption of additives during battery operation due to their inherent thermodynamic instability.

#### Artificial SEI

Another solution is to build an artificial SEI on Zn anode before cycling. Please note that the artificial SEI should function as an ionic conductor rather than an electronic conductor. Furthermore, it should provide uniform and conformal coverage of the electrode surface while demonstrating excellent chemical and mechanical stability during battery operation. They can be categorized based on their role in the zinc ion deposition kinetics: (1) accelerating the zinc ion deposition rate; (2) adjusting the solvation structure of Zn^2+^ deposition process; (3) repelling the precursors of parasitic reactions (such as SO_4_^2−^ and OH^−^).

**Fig. 14:**
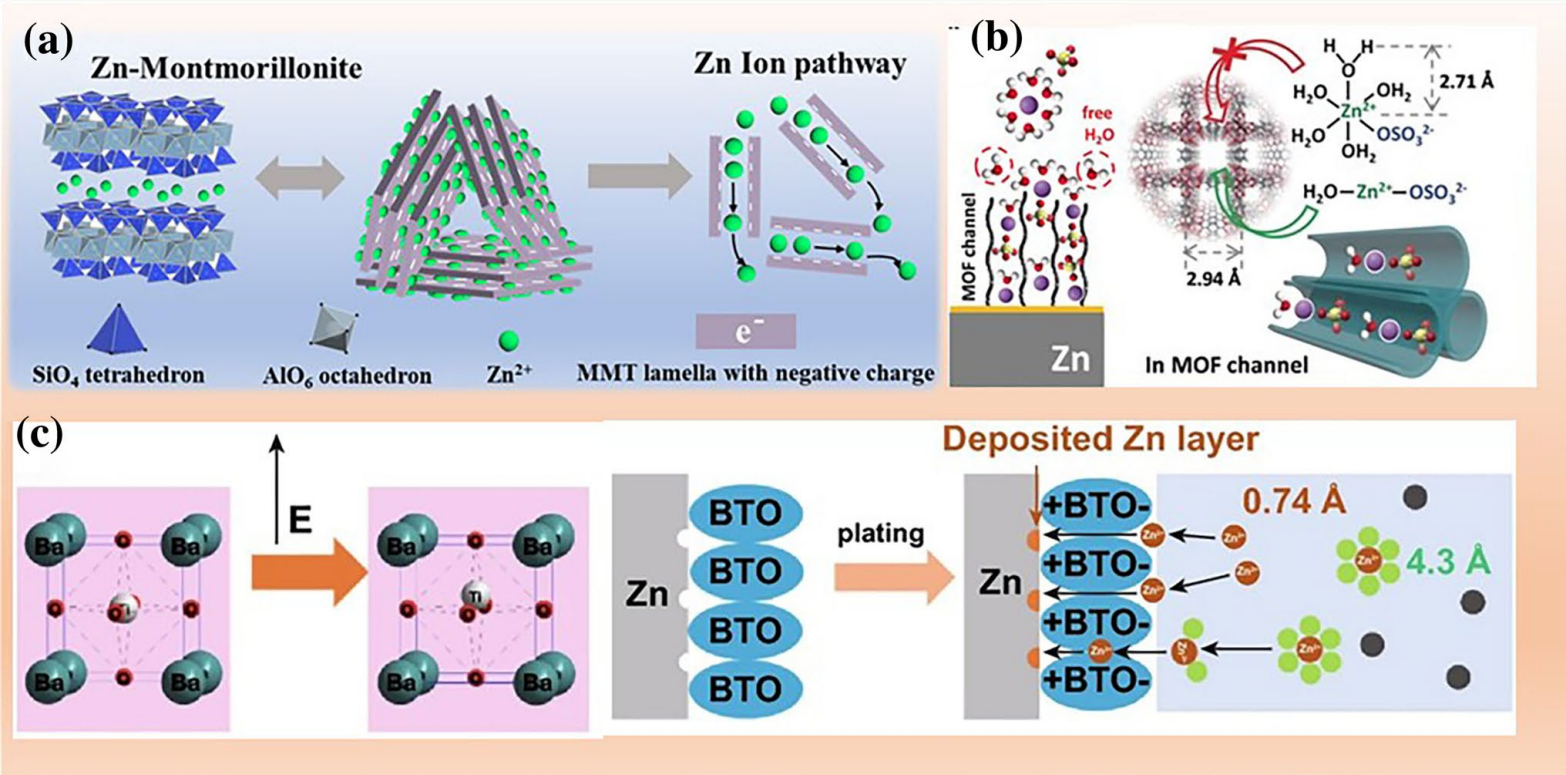
Roles of functional artificial SEI to avoid parasitic reaction. **a** Accelerating the zinc ion deposition. **b** Adjusting the solvation structure of Zn^2+^ during deposition process. **c** Repelling the precursors of parasitic reactions. **a** Zn-based montmorillonite (MMT) achieving an ultrafast zinc ion transport. Reprinted with permission from Ref. [172]. Copyright 2021 John Wiley and Sons. **b** MOF channels adjusting zinc ion de-solvation kinetics and realizing the supersaturated front surface. Reprinted with permission from Ref. [48]. Copyright 2020 John Wiley and Sons. **c** Repulsion of negative charge end of BaTiO_3_ to sulfate. Reprinted with permission from Ref. [181]. Copyright 2021 Springer

The first category of artificial SEI, which is also the most common, is characterized by being porous or having wide layer spacing (two-dimensional materials). For instance, a 2D Zn-based montmorillonite (MMT) artificial SEI is structured for Zn anode. As shown in Fig. 14a, the interlamellar of MMT provides an expressway for Zn ion transport to achieve a high ionic conductivity of 3.9 mS cm^−1^ [172]. Moreover, the dense structure of Zn-MMT film blocks the contact behavior of metal electrode with water clusters and other ions in electrolyte, realizing a high cation transference number (*t*_+_≈0.82) to inhibit parasitic reactions. Therefore, MMT-Zn anode exhibits a stable operation at ultrahigh cycling current and capacity of 10 mA cm^−2^/45 mAh cm^−2^ (77% depth of discharge). Similar to MMT, a 2D kaolin coating suppresses parasitic reactions as well as the formation of Zn dendrites by controlling the migration of Zn^2+^ ions [173]. The porous organic or organic films to regulate the migration of Zn^2+^ ions on the Zn surface are constructed. For example, nano-CaCO_3_, nano-SiO_2_, ZrO_2_, TiO_2_, and Al_2_O_3_ porous artificial SEIs physically block the side reaction precursors from the bulk electrolyte and induce the orderly migration and deposition of Zn^2+^ ions [174–177].

The second type is that some artificial SEIs not only prevent direct contact between Zn and side reaction precursors from the bulk electrolyte and regulate zinc ion deposition, but also effectively interrupt the solvation sheaths of zinc ions during deposition process, thereby reducing parasitic reactions. A recent example involves the use of a ZIF-7 coating, which possesses a small pore size of 2.94 Å, to create a supersaturated electrolyte on the surface of the Zn anode [48]. As illustrated in Fig. 14b, limited by the small tunnel of ZIF-7, large volume Zn(H_2_O)_6_^2+^ cannot pass through, and only H_2_O–Zn^2+^ ions can be released by removing the water sheath. Hence, the pre-desolvation of Zn(H_2_O)_6_^2+^ occurs before zinc deposition, significantly reducing the concentration of water near the zinc electrode and leading to an extended lifespan of up to 3000 h. A ZIF-8 coating was reported based on the same mechanism [178]. Some metal oxides and organic SEIs also assist the desolvation process of Zn(H_2_O)_6_^2+^, such as Sc_2_O_3_,^211^ ZnO,^212^ polyamide (PA) [65], and polyacrylonitrile(PAN). The Sc_2_O_3_ and 3D nanoporous ZnO SEIs can attract the water sheath of Zn(H_2_O)_6_^2+^ to lower the desolvation barrier and capture deshelled water molecules [211, 212]. In polymer artificial SEI, there are highly exposed polar functional groups on the chains (sucn as amide groups in PA and cyano groups in PAN), which can interact with the water sheath of Zn(H_2_O)_6_^2+^ to accelerate the desolvation process of zinc ions and adsorb the desolvated water molecules.

Finally, certain polar or charged artificial SEIs with a directional arrangement can effectively prevent the parasitic reaction precursors from approaching the electrode surface [179, 180]. For example, BaTiO_3_ layer is constructed on zinc electrode as an artificial SEI to suppress the side reactions via repelling the precursors of parasitic reactions [181]. BaTiO_3_, a dielectric material, can be polarized under an external electric field and the direction of polarization is parallel to the electric field [181]. As shown in Fig. 14c, under an external electric field, the Ti^4+^ can be deviated from the center of [TiO_6_] octahedral. Subsequently, directional electric dipoles induce polarized electric field on the surface. During plating process, negative charges will concentrate on the element O of Ti–O due to the surface ordered electric field of BTO. The SO_4_^2−^and OH^−^ anions will be rejected by the BTO layer due to the electrostatic repulsion; then, the side reactions would be suppressed due to the decreased collision frequency of precursors (SO_4_^2−^, OH^−^, H_2_O, and Zn^2+^). Additionally, the water molecules of Zn(H_2_O)_6_^2+^ can be attracted by the element O of Ti–O. It is possible that hydrogen bond can be constructed between the H atom of H_2_O and the O atom of Ti–O. Herein, benefiting from the switched polarization direction during charge/discharge process, parasitic reactions would be deeply restricted due to the decreased collision frequency of precursors. Moreover, the BTO layer renders considerably uniform ion pathways. Similarly, a ferroelectric polymer protective layer (poly(vinylidene fluoride-trifluoroethylene) (P(VDF-TrFE))) is coated on zinc electrode to guide zinc growth. The direction of dipoles can be regulated by pre-polarization treatment to generate negative (or positive) dipoles layer at the electrolyte-side surface [122]. Coincidentally, an inorganic–organic composite SEI consisting of Nafion and Zn-X zeolite is reported, which allows only the transmission of Zn^2+^ ions to go through the hydrophilic channels [182]. Benefitting from the negative charge framework and limited pore size of the Zn-X zeolite inorganic filler, it can repel SO_4_^2−^ and restrain H_2_O approach to Zn anode interface without affecting the passage of Zn^2+^.

It is reported that some artificial conductive SEI can effectively inhibit the zinc dendrites. However, it should be noted that in the case of Zn metal, plating the electrode surface may lead to more pronounced parasitic reactions due to the increased contact frequency between zinc metal and the electrolyte. Therefore, we will not systematically review and analyze conductive SEI.

Note that many factors determine the inhibition effect of SEI on the parasitic reaction, including pore size (or layer spacing), ionic conductivity, electronic conductivity, solubility, hydrophilicity, mechanical properties, and preparation process. By incorporating polar or charged groups or polar functional groups into the artificial SEI, which can interact with the water sheath of Zn(H_2_O)_6_^2+^ or repel precursors of parasitic reactions near the electrode interface, improved electrochemical performance can be achieved. Therefore, selecting appropriate SEI materials and optimizing the preparation process are key to enhancing performance. Moreover, compared with a single material, the combination of different materials is also an effective strategy, which can obtain more satisfactory results.

In addition to adjust the electrode/electrolyte interface, the interfaces of separator/electrode and collector/electrode also influence the kinetics of electrochemical reactions by adjusting zinc ion distribution [183, 184]. The 3D collector/electrode interface homogenizes the electric field, thereby unifying the distribution of zinc ions, accelerating the kinetics of zinc ion deposition, and inhibiting the formation of dendrites. However, the 3D interface increases the contact area between electrode and electrolyte, thereby 3D interface accelerating the occurrence of parasitic reactions from the perspective of reaction kinetics. If the separator can accelerate the flow of zinc ions and achieve uniform distribution of zinc ions at separator/electrode interface, the zinc electrode reaction kinetics is accelerated and thus, the occurrence of side reactions is suppressed. For example, dual-interface engineering (DIE)/electrode and MXene-glass fiber (GF) separator/electrode interfaces that have already been reported. The DIE/electrode interface homogenizes the ion flux and accelerates the ions transport owing to the zincophilic characteristic and spontaneous polarization effect of BTO nanoparticles. Finally, the uniform deposition of Zn^2+^ at DIE/electrode interface inhibits the growth of Zn dendrite and enhances energy storage reaction kinetics. A MXene-GF separator affords abundant surface polar groups, good electrolyte wettability, and high ionic conductivity, which is beneficial to homogenizing local current distribution and promoting Zn nucleation kinetics. It is noted that MXene-GF offers directional electrical field to expedite Zn-ion flux and repel anions. Thus, the MXene-GF separator inhibits the kinetic process of side reactions.

## Summary and Outlook

AZIBs hold great practical application prospects for large-scale energy storage due to the attributes of nonflammable, environmentally benign, high-energy density, and cost-effectiveness. However, the parasitic reactions hinder the wide application of AZIBs in energy storage systems. To address this challenge and strike a balance between the advantages and disadvantages of aqueous electrolyte, the solutions to restrain parasitic reactions can be thermodynamic and kinetic. The thermodynamic strategies relate to strengthening the inherent stability of electrolytes and zinc anode. By enhancing the thermodynamic stability, the occurrence of unwanted side reactions can be minimized. The kinetic solutions revolve around the regulation and modification of electrolyte/electrode interfaces to reduce the frequency of contact between the precursors and electrodes. By optimizing the interaction between the electrolyte and electrodes, the occurrence of parasitic reactions can be effectively mitigated. This section offers a summary and outlook on these aspects, highlighting the importance of both thermodynamic and kinetic approaches to address the challenges associated with parasitic reactions in AZIBs. By focusing on improving the inherent stability and regulating the electrolyte/electrode interfaces, the goal is to enable the wider application of AZIBs in energy storage systems.

### Enhancing Inherent Stability of Electrolyte and Zinc Anode

**Electrolyte:** The intrinsic stability of electrolyte primarily depends on solvent molecules (H_2_O) and composition of salt. So far, based on the stable anions, Zn(CF_3_SO_3_)_2_, Zn(TFSI)_2_, Zn(ClO_4_)_2_ and ZnSO_4_ as zinc salt in electrolyte endow stable CE and long life for AZIBs. Among them, the low price and high thermodynamic stable ZnSO_4_ has dominated the electrolytes of AZIBs. Regarding the solvent, strategies for strengthening the thermodynamic stability involve regulating water configuration by the addition of additives and increasing salt concentration. In detail, additives and concentrated electrolytes can reduce the presence of water clusters. Moreover, the anions and additives coordinate with Zn^2+^ to reduce the concentration of independent water molecules during the de-solvation process of hydrated zinc ions and lower the contact (or collision) between precursors and electrodes at electrolyte/electrode interfaces.

Although the high-concentration electrolytes (such as WiSE) could alleviate all parasitic reactions and expand the electrochemical stability, its high cost remains an obstacle. Adding selectable and versatile additives can not only enhance the thermodynamic stability of electrolyte by adjusting configuration of water, but also form a protective layer on the electrodes through the decomposition of additives, which is a strategy with multiple benefit. At present and in the future, more work is urgently needed to development of stable zinc salts and functional additives with reasonable prices, as well as the modification of electrolyte concentrations, to obtain thermodynamically stable water configuration and inhibit harmful parasitic reactions. Of note, the fundamental understandings of the regulatory mechanisms of water configuration in electrolyte are still very rudimentary, which should deserve more attention.

Decoupling batteries can deliver a high output voltage and energy density (Table 2), but the high price of battery equipment (especially the ion-selective membrane) limits their widespread application. Besides, the ion-selective membranes show low ionic conductivity to discount the advantages of aqueous electrolyte. Therefore, further efforts should focus on developing ion-selective membranes with low cost, higher ionic conductivity, and good mechanical strength, and designing new battery structure to promote the practicality of derivative SEI and artificial SEI.

While significant progress has been made in improving the inherent stability of electrolytes for AZIBs, further endeavors should be directed toward the following aspects in the development of future electrolytes:

1. To understand the electrolyte chemistry and zinc chemistry, it is essential to employ a combination of electrochemistry, spectroscopy, microscope, theoretical calculations, and simulations.
2. As we all know, the thermodynamic stability of solvent is depended on the O−H bond in water molecules. It has important theoretical and guiding values to study the relationship between the configuration structure of water molecules and the strength of O–H bond. The electrolyte composition (including additives, concentration, anion, and cation) influences the configuration of water molecules, thus affecting the strength of O−H bond in H_2_O molecules and creating inert water environment. For the future development of electrolytes, attention should be paid on the regulatory of water configuration, as well as the interplay between electrolytes composition and electrodes.
3. In the decoupling systems, it is necessary to reveal the thermodynamic stability of electrodes in different chambers (environments). Specifically, the potential of redox couples in each chamber is affected by the ionic configuration and concentration. How to ensure the electrode with high thermodynamic stability under a high output potential of the decoupling battery is the focus of future research.
4. We consider that the parasitic reactions caused by water molecules is inevitable in AZIBs because of the water sheath of zinc ion. The de-solvation process of hydrated zinc ions to yield highly active water molecules at electrode interface should be taken seriously, unless the water sheath of zinc ion is completely replaced by other non-water molecules. Additives with high Gutmann donor number and cosolvent to replace the water sheath should be developed. Thus, little or no independent water molecules accumulate at electrode/electrolyte interface during de-solvation process. In addition, future work should also pay attention to water content to balance the ion diffusion, ionic conductivity, and energy barrier of removal sheath of Zn^2+^.

**Fig. 15:**
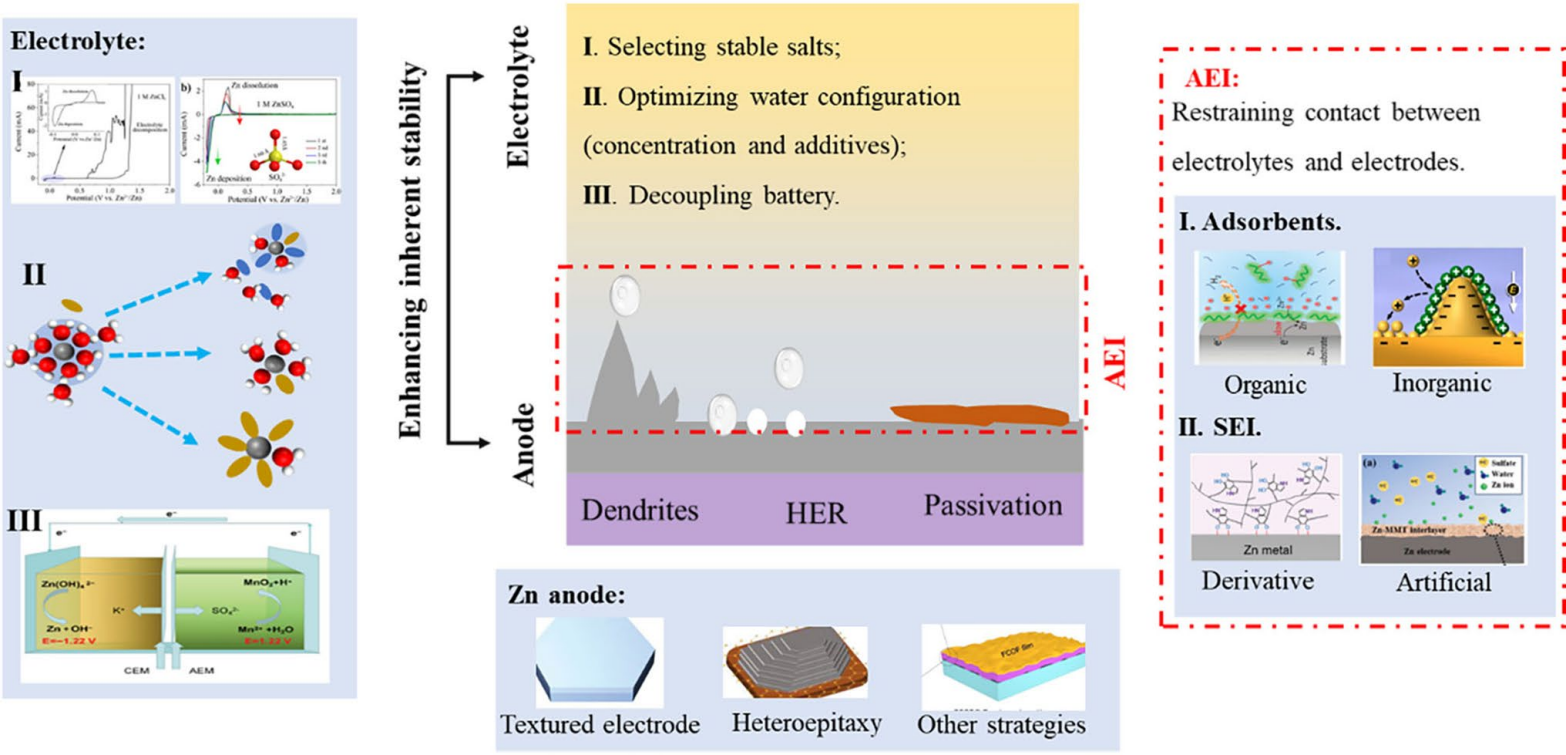
Overview of modification strategies for future anode and electrolyte to suppress parasitic reactions at zinc side. These reactions are directly or indirectly caused by electrolyte. The developed strategies can be categorized into thermodynamic and dynamic strategies. The thermodynamic strategies are to enhance the inherent thermodynamic stability of zinc anode and electrolyte. The dynamic strategies are to avoid (or reduce) the contact between precursors (such as H_2_O and anion) and electrodes, that is, to regulate or modify the electrolyte/electrode interfaces

**Zinc anode:** The different crystal facets endow different physical and chemical properties as wells as differential thermodynamic stability. Benefiting from the Zn(002) as the densest packing surface, it shows minimum surface free energy, which is conducive to resisting parasitic reactions on zinc anode, especially showing strong corrosion resistance and dendrite inhibition potential. In this review, we summarize the strategies and corresponding principles to achieve highly exposed Zn(002) deposition from the perspective of improving the stability. As shown in Fig. 15, the strategies include (i) directly employing single crystal or highly textured zinc with exposed Zn(002) as anode; (ii) constructing an artificial substrate possessing a low lattice mismatch ratio with Zn(002) for heteroepitaxial growth; (iii) enhancing the adsorption energy and reducing the diffusion energy barrier of Zn atoms at Zn(002) by artificial SEIs, additives, and separators. Although much progress has been made in Zn(002) textured anode, there are still many challenges for the wide application. Here, we put forward some personal views on the thermodynamic strategy to develop zinc anode:

1. Constructing highly (or only) exposed Zn(002) anode is the most direct and beneficial thermodynamic strategy to stabilize zinc anode and avoid parasitic reaction. Therefore, the focus in the future is to modify the commercial zinc metal. Although the rolling, etching, and thermal annealing techniques were employed to achieve highly exposed Zn(002) anode, it cannot be ignored that these treatment technologies may increase the internal energy of Zn crystal, such as stress brought by the rolling process; lattice defects brought by etching, thermal annealing brought high energy grain boundaries. It is believed that more researchers pay attention to look for new preparation processes to alleviate or release the increased internal energy of zinc crystal.
2. The increased lattice distortion energy of deposited zinc cannot be ignored due to the lattice mismatch of substrate with the Zn facet. Therefore, more in-depth studies based on elimination of lattice distortion energy are necessary.
3. There is still a lack of deep understanding on the adjusting zinc ion deposition along thermodynamically stable facets. These strategies are considered to eliminate the initial deposition trend along Zn(100) and Zn(101) by adjusting the adsorption energy and diffusion energy of Zn atoms, and interface energy of exposed facet through monotony theoretical calculation and simulation, which is very unreliable. Therefore, it is valuable to combine experiments to reveal the mechanism of the deposition.
4. Although there are many means to monitor and characterize the stability of zinc electrode, it is urgent to dictate the protocol for evaluating the thermodynamic stability of zinc electrode. The observed phenomena and experimental data are related to many factors, such as electrolyte, separator, current density, capacity, voltage, and temperature. Therefore, a standard protocol would be helpful to evaluate the thermodynamic stability of the zinc anode.

### Restraining Parasitic Reactions Dynamics at Zinc/Electrolyte Interface

If the occurrence of parasitic reactions is inevitable from a thermodynamic perspective, it becomes crucial to minimize their occurrence from a dynamic standpoint. As these reactions take place at the surface of the electrodes, it is possible to manipulate the reaction sites to reduce the reaction rate. The main idea is blocking the contact area (or sites) between water and electrodes.

The modification of AEI involves two main strategies: adsorbent strategies and the construction of SEI (Fig. 15). From a dynamic perspective, adsorbents can block active sites through electrostatic forces, functional groups, and other physical effects. The advantage of this strategy lies in its controllability and intelligent selectivity. However, the discontinuous distribution and weak binding force on the electrode surface lead to weak inhibition of side reactions. Therefore, it is necessary to balance many factors when designing electrode adsorbents.

The construction of AEI, including derivative SEI and artificial SEI on Zn anode before cycling, can physically separate parasitic reaction precursors while allowing the passage of Zn^2+^. The porosity and composition of SEI can be designed to further optimize the de-solvation process of hydrated zinc ions. In our opinion, non-conductive inert artificial SEI is the optimal choice. A conductive protective layer can lead to the deposition of zinc on the protective layer, triggering severe parasitic reactions due to the contact between zinc and electrolytes. Whether adsorbents or SEI, factors such as thermal, chemical, and electrochemical stability, as well as adhesion on the zinc metal, should be taken into consideration.

The design of AEI can be carried out from the perspective of controlling other factors, such as the selection of concentrated electrolytes and textured electrodes, and the preparation of functionalized separators that uniformly transfer ions, in addition to artificial SEIs and derivative SEIs. Therefore, a stable AEI can be constructed from multiple perspectives.

Here, some perspectives and the future development of the Zn/electrolyte interface are provided.

1. The thickness of AEI causes an opposite relationship with the inhibition effect of parasitic reaction and the influence on ionic conductivity, so a balance needs to be made between them.
2. The SEI derived from additives should exhibit good environmental friendliness, low or no toxicity, and good safety and stability. The durability and mechanical properties of derivative SEI are two key factors to be considered.
3. Understanding the active site of the zinc anode is of utmost importance in guiding the minimal usage of adsorbents. Exploring the interactions among the electrolyte, adsorbent, and electrodes will be crucial for unraveling the underlying mechanism. Specifically, studying the impact of adsorbents on the solvent sheath of zinc ions and the de-solvation process holds significant value.
4. At electrolyte/electrode interface, the unpredictable chemical environment evolution perplexes researchers during AZIBs operation. It is very necessary to understand and capture in real time at the real location the chemical environment and corresponding solvated structure during charging and discharging. Future work should concern the advanced characterization techniques to track interface chemistry.

Great efforts have been made to overcome parasitic reactions, but there is still a long way to go before practical application. In this review, we have summarized the reported strategies from the perspective of thermodynamics and dynamics, respectively, in fact, but these strategies interact in thermodynamics and dynamics to inhibit parasitic reactions. For example, in WiSE, not only the O–H strength in water molecule is enhanced from the thermodynamics, but also the contact frequency between water and electrode is reduced from dynamics. The future solutions are to exploit the strategy to enhance the intrinsic stability of battery modules, while lowering contact (or collision) frequency between precursors and electrodes at electrolyte/electrode interfaces. These strategies not only inhibit these parasitic reactions, but also do not cause other problems, such as safety problems and short battery life. In our opinion, the low-price concentrated electrolytes have the potential to improve the performance of AZIBs and overcome parasitic reactions. In the strategy exploration, we can also borrow technologies and solutions from other battery fields to solve these parasitic reactions. At the same time, theoretical simulation methods and advanced characterization technology should be further developed to understand and control parasitic reactions. Through the unremitting efforts, AZIBs will be industrialized and occupy the energy storage market.
